# Miniaturization of CMOS

**DOI:** 10.3390/mi10050293

**Published:** 2019-04-30

**Authors:** Henry H. Radamson, Xiaobin He, Qingzhu Zhang, Jinbiao Liu, Hushan Cui, Jinjuan Xiang, Zhenzhen Kong, Wenjuan Xiong, Junjie Li, Jianfeng Gao, Hong Yang, Shihai Gu, Xuewei Zhao, Yong Du, Jiahan Yu, Guilei Wang

**Affiliations:** 1 Key Laboratory of Microelectronics Devices & Integrated Technology, Institute of Microelectronics, Chinese Academy of Sciences, Beijing 100029, China; hexiaobin@ime.ac.cn (X.H.); zhangqingzhu@ime.ac.cn (Q.Z.); liujinbiao@ime.ac.cn (J.L.); xiangjinjuan@ime.ac.cn (J.X.); kongzhenzhen@ime.ac.cn (Z.K.); xiongwenjuan@ime.ac.cn (W.X.); lijunjie@ime.ac.cn (J.L.); gaojianfeng@ime.ac.cn (J.G.); yanghong@ime.ac.cn (H.Y.); gushihai@ime.ac.cn (S.G.); zhaoxuewei@ime.ac.cn (X.Z.); duyong@ime.ac.cn (Y.D.); yujiahan@ime.ac.cn (J.Y.); 2 Microelectronics Institute, University of Chinese Academy of Sciences, Beijing 100049, China; 3 Department of Electronics Design, Mid Sweden University, Holmgatan 10, 85170 Sundsvall, Sweden; 4 State Key Laboratory of Advanced Materials for Smart Sensing, General Research Institute for Nonferrous Metals, Beijing 100088, China; 5 Fert Beijing Institute, Big Data Brain Computing (BDBC), Beihang University, Beijing 100191, China; cuihusan@ime.ac.cn; 6 School of Artificial Intelligence, University of Chinese Academy of Sciences, Beijing 100049, China; 7 School of Microelectronics, University of Science and Technology of China, Anhui 230026, China

**Keywords:** FinFETs, CMOS, device processing, integrated circuits

## Abstract

When the international technology roadmap of semiconductors (ITRS) started almost five decades ago, the metal oxide effect transistor (MOSFET) as units in integrated circuits (IC) continuously miniaturized. The transistor structure has radically changed from its original planar 2D architecture to today’s 3D Fin field-effect transistors (FinFETs) along with new designs for gate and source/drain regions and applying strain engineering. This article presents how the MOSFET structure and process have been changed (or modified) to follow the More Moore strategy. A focus has been on methodologies, challenges, and difficulties when ITRS approaches the end. The discussions extend to new channel materials beyond the Moore era.

## 1. Introduction

In 1965, Gordon Moore, the founder of Intel, published his famous paper describing the evolution of transistor density in integrated circuits. Although his first insight was to establish a business roadmap to increase the profit of the company, he later built the fundaments for technology roadmap in the semiconductor industry. Moore’s idea was based on doubling the transistor density in the chip every 18 months, which causes the transistors to become smaller in size and consumes lower power while performing at higher speed [1]. 

With years of continuing MOSFETs (metal oxide effect transistor) down-scaling, different non-ideal factors e.g., short channel effects (SCEs), poor electrostatics integrity, and large device variability appeared. Therefore, conventional bulk FinFET and fully depleted silicon on the insulator (FDSOI) are proposed to improve the above problems by applying low gate voltage to fully deplete the ultra-thin silicon [2]. Currently, bulk FinFET has been widely used in mass production from 22 nm to 10 nm node and will be extended to the 5-nm node [3,4,5,6]. In fact, the critical dimension (CD) of the device, e.g., gate length (*L_g_*), applied voltage (V_DD_), and effective oxide thickness (EOT) are not strictly scaling, according to the Moore’s law. The foundries seek an improvement of driving current (I_DS_) at the same leakage or achieve the smaller leakage at the same I_DS_. On the approach to the end of the technology roadmap, the 3-nm node and the traditional bulk FinFET technologies would suffer from enormous challenges [7]. Thus, new device structures, new materials, and new integration approaches have to provide new solutions. Therefore, novel promising device architectures like fin-on-insulator (FOI) FinFET [8,9,10,11], scalloped fin FinFET [12], nanowire (NW) FETs, and the stacked NW device [13,14,15] have demonstrated great improvement for short channel effects (SCEs), leakage control, and higher electron and whole mobility. The fin-on-insulator (FOI) FinFET, fabricated on the bulk Si substrate with a special process takes both advantages of bulk FinFET and SOI technologies. Therefore, it may be one of the most promising candidates for further device scaling. In addition, the low cost and fully Metallic Source and Drain (MSD) process is extensively investigated for the FOI FinFET [9].

Other architecture such as scalloped fin FinFETs with mainstream all-last HKMG (high-*k* and metal-gate) technology could provide a larger control area and obtain a great improvement for SCEs. Stacked gate-all-around (GAA) NW or nano-sheet is also receiving increasing attention among all device structures. This is considered to be the most promising candidate beyond FinFET technologies for a 3-nm node due to its special characteristic, such as quasi-ballistic transport, steep sub-threshold slope, and one-dimensional channel geometry [13,14].

3D-monolithic or 3D sequential CMOS technology is based on stacking active device layers on top of each other with very small 3D contact pitch (similar pitch as a standard contact) [16,17]. This approach could achieve a 14-nm circuit performance by using 3D sequential CMOS technology with lower parasitic resistance, capacitor, and signal delay. In addition, this integration scheme offers a wide spectrum of applications including (i) increasing integration density beyond device scaling, (ii) enabling neuromorphic integration where RRAM is placed between top and bottom tiers, and (iii) enabling low-cost heterogeneous integration for e.g., smart sensing arrays. However, such an integration process faces the challenges of fabricating high-performance devices in the top tier without degrading the electrical characteristics of the bottom tier [18,19]. 

The CMOS scaling-down in process, V_DD_, and temperature (PVT) are becoming a major issue for the nanoscale IC design. The need for low power induces supply voltage scaling, which makes voltage variations a significant design challenge. Moreover, the operation frequency is sensitive to die temperature variations. Therefore, it is increased at high junction temperatures. It is known that process variations are a serious concern due to uncertainty in the device and interconnects characteristics. Process variations negatively impact the speed, stability, and power consumption of traditional transistor designs. 

With the continuing scaling of devices, the driving current would become bigger and the frequencies of transmitted signals become higher [20,21].

This article presents how the technology roadmap deal with miniaturization of CMOS including advanced lithography for patterning nano-scaled transistors, process integration, (wet and dry) etching, strain engineering with an emphasis on SiGe epitaxy for source/drain (S/D), dopant implantation, gate formation including deposition of high-*k* material, and the metal gate using the atomic layer deposition (ALD) technique, and III-V materials for high carrier mobility in the channel for FinFETs. The discussions have a focus on the challenges and difficulties of the path of More Moore and even provide a glimpse of the beyond Moore era for CMOS. 

## 2. Miniaturization Principles

**Figure 1:**
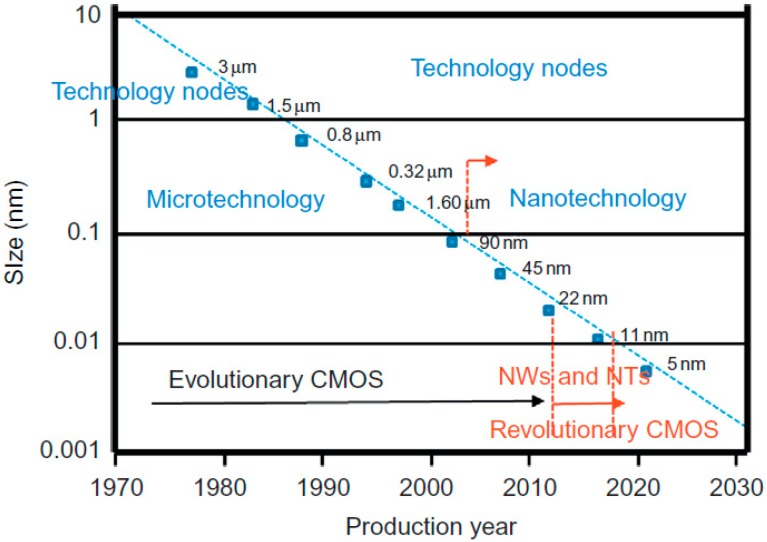
Miniaturization of the transistor gate length in different technology nodes and production years [22].

Figure 1 shows the official technology roadmap, which was originally established in the early 1970s and the semiconductor industries began to down scale the transistors [22]. In 2003, when the transistor size shrunk to sub 100 nm, the nano-electronic era was inaugurated. The continuation of down-scaling lead to the parasitic capacitance and the resistance increased. Lastly, the 2D transistors were abandoned and 3D FinFETs were introduced. This is considered as a revolutionary design in the transistor world, which paved the path for sub 22 nm FinFETs with high performance and full control on the carrier transport in the channel.

The down-scaling of the transistors results in operation at lower supply voltages as well as switching with less current.

**Figure 2:**
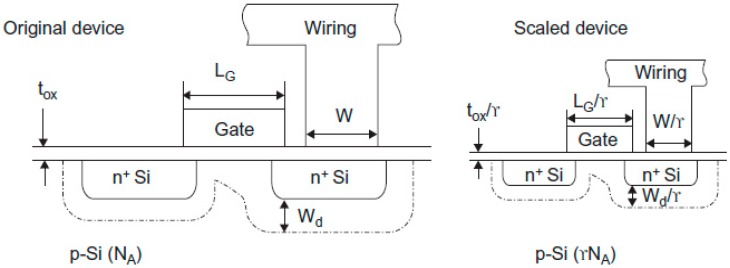
A schematic drawing of MOSFET downscaling [23].

On one hand, the shorter channel causes lower gate capacitance and higher drive current resulting in faster transistors. On the other hand, the shorter channels contribute to higher S/D and Gate leakage since gate oxide becomes thinner. The smaller transistors have both lower V_DD_ and threshold voltage (V_T_) or, in principle, lower dynamic power is obtained. The main rules to miniaturize MOSFET with a factor of γ is demonstrated in Figure 2 [23]. This task is performed for transistors when the gate length and width, oxide thickness, junction depth, and substrate doping are downscaled. Therefore, both supply and threshold voltages are also scaled by a factor of γ. In this way, the electric field is maintained constant. Meanwhile, the density of transistors is increased by factor of γ^2^. In this design, the ratio between gate length and width is also unchanged.

However, information on the size of the pitch in nanometer technologies and the freedom in choosing the size of the transistors can be different. Simply, the pitch parameter may not follow the same trend as the general miniaturization of technologies.

## 3. Lithography of Nano-Scaled Transistors

A state-of-the-art lithography seeks sharp patterns with high reproducibility. For 20-nm and 14-nm node technology, 193 nm ArF immersion with multiple patterning has been mainly used [24]. Meanwhile, 193-nm immersion with self-aligned double patterning (SADP) and self-aligned quadruple patterning (SAQP) techniques is intended to be used for a 7-nm technology node [24].

SADP is a technique which applied spacer transfer process for small pitch whereas SAQP is applied twice self-aligned double patterning to develop very narrow features [25].

There is a strong effort to apply extreme ultraviolet (EUV) lithography and 193 nm immersion with multi-patterning for a 7-nm node. Although EUV simplifies the patterning process for the 7-nm node, EUV still has issues with resists and mask infrastructure as well as the power source, which have to be solved before high-volume manufacturing.

**Figure 3:**
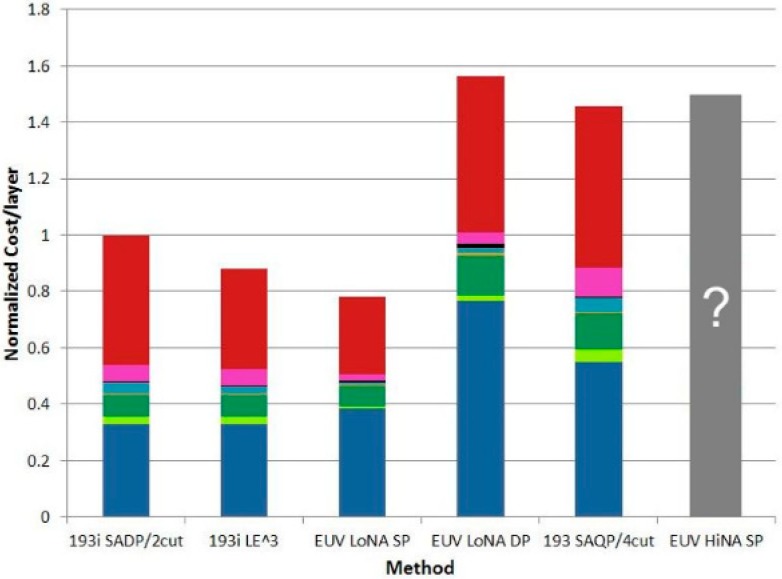
Normalized cost/layer vs. lithography method.

Figure 3 indicates that the lithography cost depends on the layer and, therefore, the cost of applying either 193i triple patterning or 193i SADP are roughly equal to single-patterning with EUV. This means that the choice of the lithography method depended more on the performance-involved trade-offs [26]. For 7-nm and 5-nm nodes, there is a risk that quad-patterning may occur from 193i and double-patterning from existing EUV tools, or single-patterning from as-yet undelivered high numerical aperture (NA) EUV tools.

To apply 193 nm immersion lithography with multiple patterning for 7-nm and 5-nm nodes has difficulties. The main problem is overlay, which involves the ability of a scanner to align the various mask layers accurately on top of each other. There may be too many masks at each new node. This slows down throughput in the mask shop, increases the possibility that errors will be introduced, and raises the cost at the same time. 

Recently, it has demonstrated excellent industrialization progress of its pellicle, with tests confirming that pellicles can withstand 245 W source power and an offline power lifetime test indicating 400 W capability. Compared to the 7-nm logic node, the requirements for EUV masks is tighter for 5 nm. Meanwhile there is good progress to support 5 nm in areas such as reducing mask blank defects [27,28].

The EUV mask infrastructure is the need to manufacture defect-free photo masks where an actinic mask review capability is a critical success factor [29,30]. The ongoing development for anEUV pellicle solution alleviates industry concern about one significant source of line-yield risk [31]. Because pellicles are currently unavailable for EUV lithography, other measures need to be taken to deal with contamination that can occur during mask transport and usage. Such contamination can indeed occur, and it has been observed by practitioners of EUV lithography. This may occur with sufficient frequency, which justifies the concern for repeating defects that can reduce the yield significantly [32].

In addition to the high EUV sensitivity, low local CD uniformity, and high patterning resolution, the next generation resist systems should also efficiently solve the issues of pattern collapse, resist homogeneity, etch resistance, UV out of band, outgassing, high volume manufacturing (HVM) compatibility, defects, and shelf-life [33].

To improve throughput in HVM, the resist sensitivity to the 13.54-nm wavelength radiation of EUV needs to be improved while the line-width roughness (LWR) specification must be held to low single-digit nm. With a 250 W source and 25 mJ/cm^2^ resist sensitivity, an EUV stepper should be able to process ~100 wafer-per-hour (wph), which should allow the affordable process when matching with other lithography technologies.

**Figure 4:**
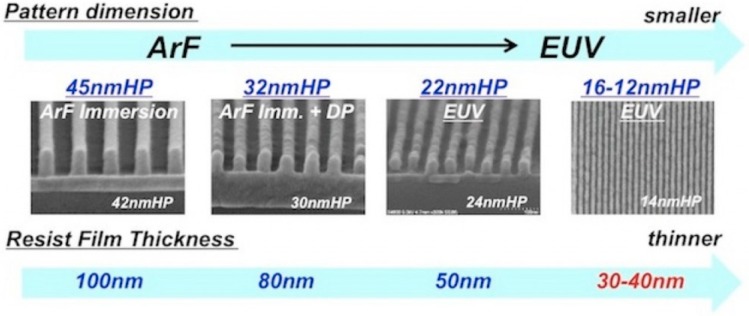
Evolution of the lithography technique where the pattern becomes denser and smaller in each new technology node. To prevent pattern collapse, the thickness of resist is reduced proportionally to the minimum half-pitch (HP) of lines/spaces.

Figure 4 shows that higher absorption allows the use of thinner resist, which mitigates the issue of line collapse. Resistance as thin as 18 nm has been patterned over a 70-nm thin Spin-On Carbon (SOC) layer without the need for another Bottom Anti-Reflective Coating (BARC).

## 4. Process Integration of New Transistor Architecture

### 4.1. Process Flow of 2D and 3D Transistors

**Figure 5:**
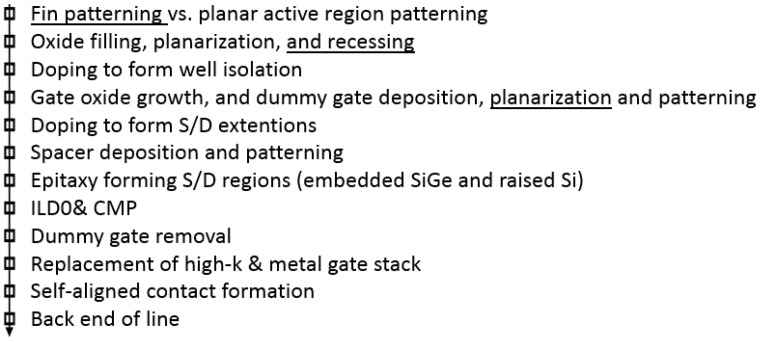
Process flow for the bulk FinFETs or planar transistors. The FinFETs process are underlined [36].

The process flow of FinFETs consists of the SADP technique, followed by oxide filling, planarization using CMP (chemical mechanical polish), and etching to reach the fin active region and form shallow trench isolation (STI).Afterwards, the process flow is similar to the planar transistors e.g., well doping, dummy gate deposition and patterning, spacer formation, SiGe-epi, and S/D formation, interlayer dielectric zero (ILD0), chemical mechanical polish (CMP), dummy gate etch, high-*k* and metal-gate (HKMG) process, self-aligned contacts (SACs) for silicide and metal formation, local interconnects (LI), and back-end-of-line (BEOL) interconnect construction [34,35,36], as shown in Figure 5.

**Figure 6:**
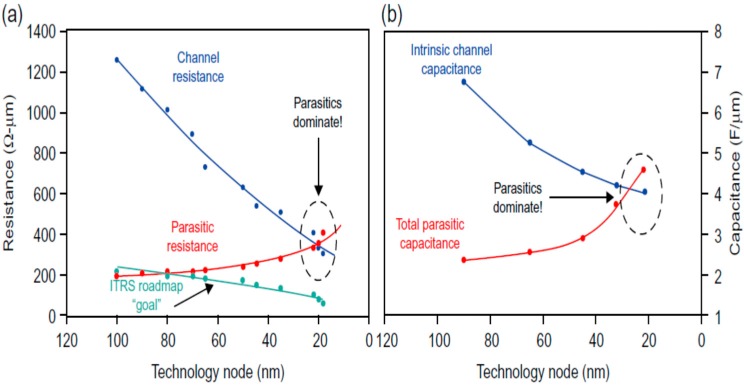
(**a**) Parasitic resistances and (**b**) capacitances in each technology node [22].

On the path of CMOS miniaturization, the smaller contact size leads to higher contact resistance and contact-to-gate capacitance. Normally, the parasitic effects had no big impacts on the transistor performance because they were significantly smaller than the resistance and capacitance of the channel. However, both these effects becomes proportional to the gate length, which has been significantly reduced during past years. The parasitic effects became comparable or even larger than the intrinsic channel (gate and body) capacitance and resistance, as is shown in Figure 6.

**Figure 7:**
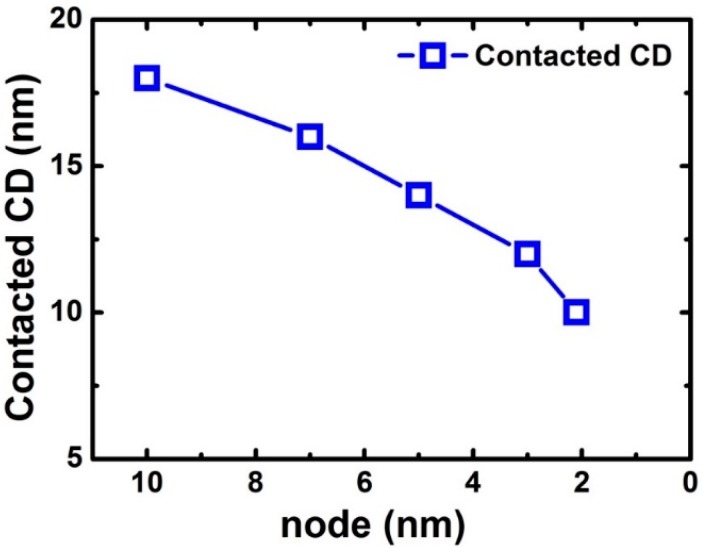
Values of contacted CD for advanced device.

The contacted CD of FinFET or lateral GAA NW device is 18 nm for the 10-nm node and the contacted CDs are expected to be 16 nm, 14 nm, and 12 nm for 7 nm, 5 nm, and 3 nm in the future, respectively [37], as is shown in Figure 7. The contacted resistivity has reached sub 10^−9^ Ω·cm^2^ for advanced CMOS FinFET beyond 7 nm, and the values need to be smaller in the future [38].

### 4.2. Challenges in FinFETs’ Process

**Figure 8:**
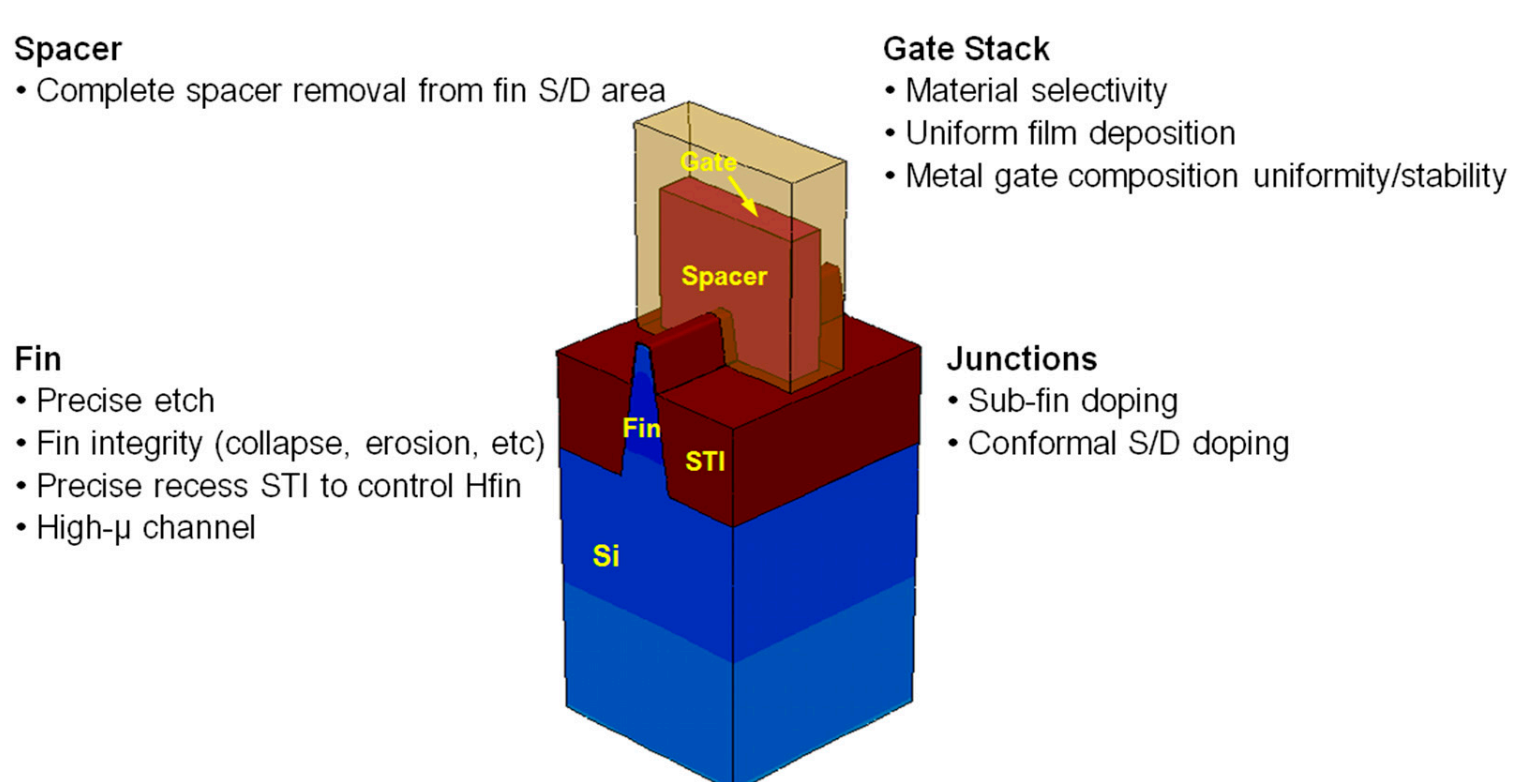
New challenges in the miniaturization of FinFETs [36].

Electrical characteristics of FinFETs are related to the fins’ profile and dimensions [39]. In order to transport higher current, longer fins are required, which leads to several challenges to manufacture transistors, as shown in Figure 8.

The process of long Si fins creates difficulty for the integration of poly gate, spacer, and replacement metal gate. One of the problems’ roots is not easy to etch the poly gate with a high aspect ratio [39]. Charging and micro-loading of etching results in variable *L_g_*. An over-etch process is needed to clean the poly residual, and to remove the offset spacers on the fin-sidewalls [40,41]. Unfortunately, both these over-etchings result in damages of the Si fins. A remarkable Si loss may occur after wet cleaning and the solution has to be more diluted and should be used at low temperatures. Therefore, the dry and wet etching need more attention to be optimized to produce 3D gates with minimum *L_g_* variation and Si loss in fins.

The patterning of fins is performed by SADP [42]. In this process, the depth of fin etching is usually determined by time. Meanwhile, the fins located at the edge of the wafer may show larger profile variation compared to those in the middle. To obtain uniform fin dimensions, dummy features could be used [43]. In this case, some dummy fins are necessary to be cut at the pitch. It is important to mention that, when the fin pitch shrinks and becomes compatible to the overlay limit, cutting fins becomes more difficult. Other steps of the FinFET’s process, e.g., fin isolation by STI and channel-stopper doping become more challenging, because the tighter pitch makes it more difficult to control the STI profile as well as doping variation.

In conclusion, in order to maintain the integrity of fins with a high aspect ratio is a challenging task [32]. The dry etching of fins is not a straight forward task because of the 3D topography. Thus, a plasma pulsing scheme is viable to decrease Si loss [39]. In a similar way, the oxidation of fins is a non-uniform process and it is faster at corner and tip areas.

One of the important issues in the FinFETs process is doping the fins [44,45]. A conformal doping profile in the S/D and extension regions has to be performed to create uniform carrier conduction in the fin-channel. The tight pitch of the fins restricts the incident beam angle in ion implantation (I/I), and may result in shadowing neighboring fins.

During the I/I, the Si fins become amorphous and, later, an annealing treatment is applied for re-crystallization. Unfortunately, this thermal treatment usually leads to poor dopant activation and formation of defects [44]. The poor fin quality has strong impact on the epitaxial quality of SiGe in S/D epitaxy as well as contact resistance in those regions. An increase of Si wafer temperature during I/I could be an appropriate solution to decrease the amorphous depth and fin damage [44,45]. Several reports have presented different innovative doping methods. Solid-source doping, molecular monolayer doping, and conformal plasma doping can improve the doping profile. 

## 5. SiGe Epitaxy of Nano-Scaled Transistors

SiGe was integrated in S/D regions as stressor material for the first time in the 90-nm technology node to induce uniaxial strain in the channel region. Selective epitaxy growth (SEG) of SiGe was applied to fill out the recessed S/D regions. The advantage of uniaxial strain to the biaxial one is higher carrier mobility even for high electric fields. The embedded SiGe in S/D regions has been continuously increased from 19% to 45% when the transistors were miniaturized from 90-nm to 22-nm nodes [46,47,48,49,50]. In a 45-nm node, the shape of recess in S/D regions was modified from a round shape to sigma shape in order to further increase the SiGe strain since the layers could be located closer to the channel [51,52,53]. 

**Figure 9:**
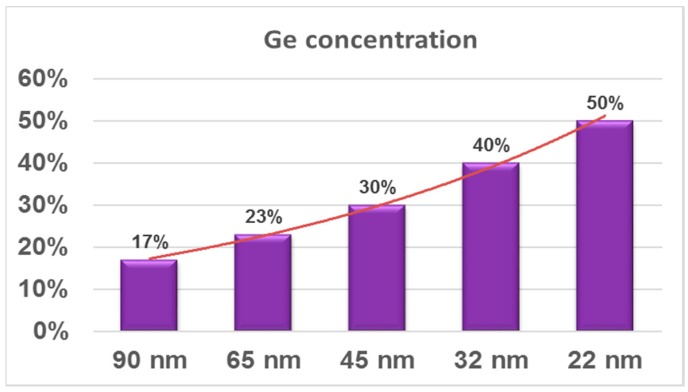
Ge contents in S/D for different technology nodes.

Beyond the 22-nm node (3D transistors), the SiGe layers are being grown on the Si fin to raise the S/D regions. The summary of Ge contents in S/D regions for different technology nodes is shown in Figure 9.

The SEG of SiGe on Si-Fin and nanowires has its own complexity and challenges. The SiGe growth may suffer from a series of problems: facet formation [54,55], defects, micro-loading, non-uniform strain distribution, surface roughness, and the pattern dependency effect [56,57,58,59,60]. Among those problems, the pattern dependency effect occurs when the density and size of the transistor vary in a chip. The reason for the pattern dependency of SEG is mainly non-uniform consumption of reactant gas molecules when the exposed Si area varies in a chip. Hence, more careful optimization of the growth parameters as well as redesigning chip layout for uniform exposed Si areas could create uniform gas consumption during epitaxy for a successful process [56,57,58,59,60].

Currently, there is a strong attitude to replace the Si channel with SiGe or Ge in FinFETs with a high aspect ratio. The idea behind is to terminate the formed defects close to the fins’ sidewall during epitaxial growth. This method is also called high aspect ratio trapping (ART) and by using it, high-quality film in the vertical direction is obtained.

**Figure 10:**
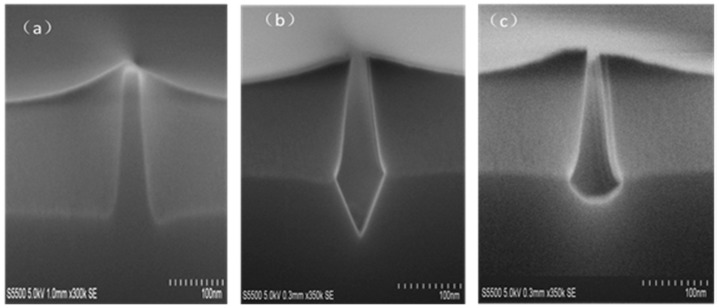
(**a**) Si fin covered with SiO_2_ (**b**) removal of Si in the fin after wet-etch by TMAH and (**c**) HCl vapor etch [61].

There are two methods to remove the Si and form the trench including the wet etch using Tetramethylammonium hydroxide (TMAH) and the vapor etch using HCl inside the CVD (chemical vapor deposition) chamber. The biggest difference between these two methods is the control of the silicon morphology at the bottom of the trench. A “V-shape” (111)-oriented of Si crystal is formed in the TMAH etch. Meanwhile, the HCl vapor method offers (311)-oriented facets of Si at the bottom of the trench, as shown in Figure 10a–c.

**Figure 11:**
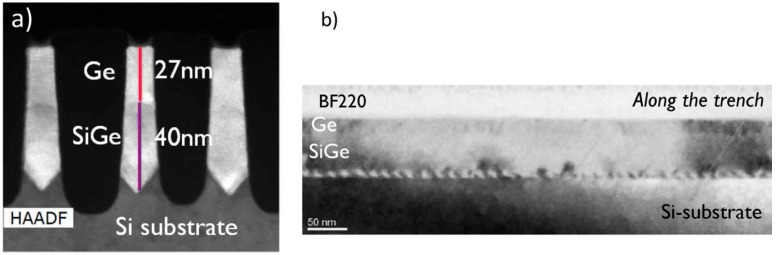
Cross-section TEM of strained Ge-cap/ SRB Si_0.3_Ge_0.7_ grown in an oxide trench and observed at (**a**) fin cut and (**b**) along the trench. The Si was removed by the wet etch prior to epitaxy [62].

After filling the fins, the Chemical Mechanical Polish (CMP) technique is used to planarize the lateral overgrown part. Later, the STI oxide is removed by diluted HF solution to expose the Ge or SiGe fin. Figure 11 is the process scheme of SiGe or Ge SEG in the channel region in a FinFET structure. The process initiates to form a “V-shaped” Si recess and growth of the strain relaxed buffer (SRB) of Si_0.3_Ge_0.7_ [62].

For a better control over a short channel effect, a more aggressive design e.g., the Gate-All-Around (GAA) structure is proposed to be integrated in the near future. For such transistors, SiGe or Ge are proposed to be grown as channel material for high mobility.

**Figure 12:**
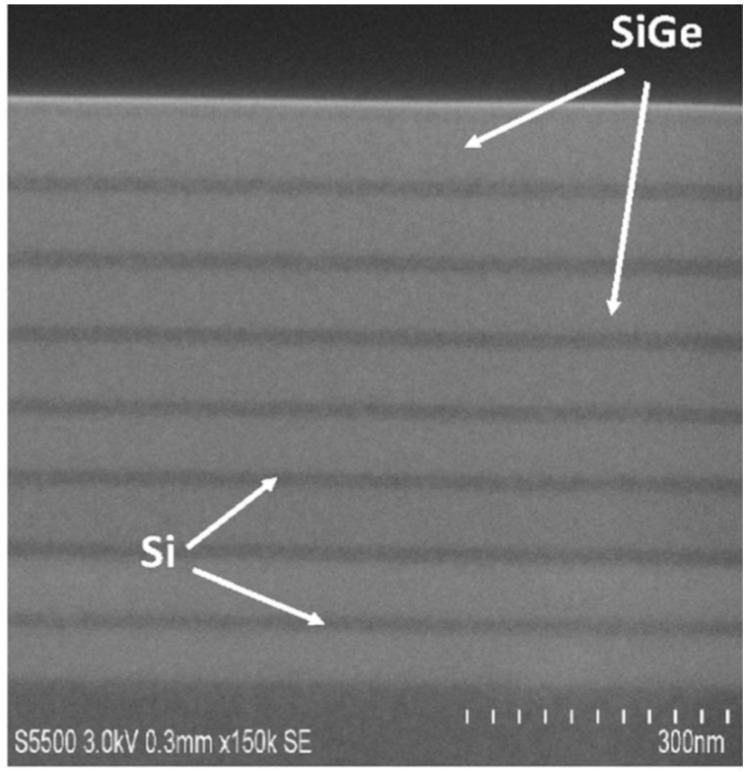
HRSEM of a multilayer of the SiGe/Si structure with eight periods [66].

For the sub 10-nm technology node and beyond, the nano-wire device might be one of the promising candidates to obtain better gate control and lower leakage current [63,64,65,66]. In this approach, SiGe/Si multi-layers are grown where either SiGe or Si can be etched selectively to form channel regions for NWs. Figure 12 illustrates an image of a multilayer SiGe/Si structure with eight periods for forming vertical NWs.

## 6. Monolayer Doping

Monolayer doping (MLD) is a self-assembly doping process, which can be applied for doping NWs. This doping method is dominated by a surface chemical reaction between the semiconductor substrate and dopant-containing organic molecules [67,68,69,70,71]. Compared to conventional implantation, MLD introduces fewer defects into the substrates [72,73,74,75] and the dopant-containing molecules could attach uniformly on the surface, which results in a better conformal doping profile [73]. 

**Figure 13:**
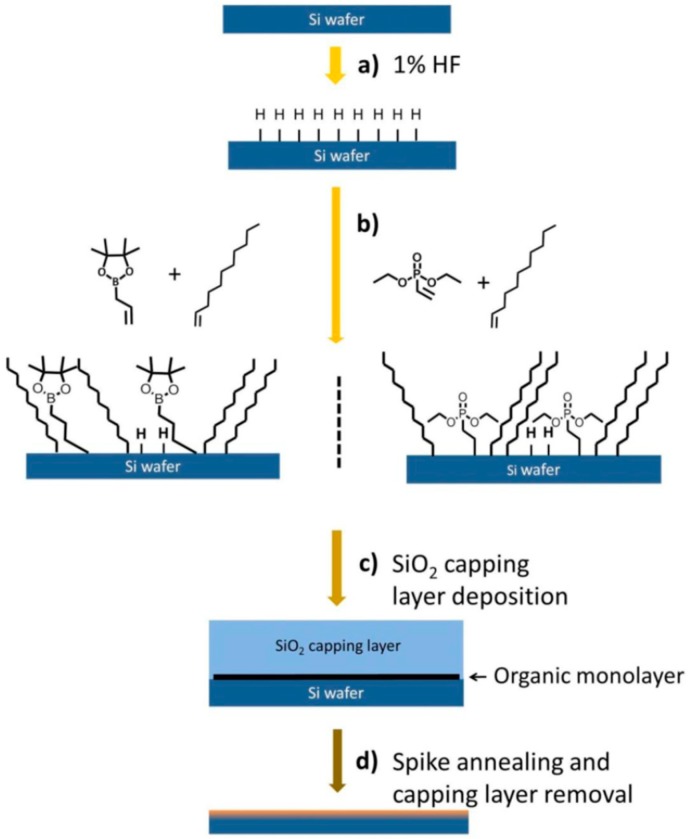
Schematic of the monolayer doping process [70].

The basic procedure of MLD for nano-sheets is shown in Figure 13 [70]. However, the process could be easily used for NWs. The solution aqueous HF or NH_4_F was first used to remove the native oxide on the surface and to obtain a hydrogen-terminated surface. Later, the substrate will be immersed into dopants containing liquids or solution of organic materials [70,71,72,73,74,75]. The organic material could be dopant atoms contained in alkene or alkyne. After that, a low temperature treatment or light irradiation will be adopted to enable a reaction, which is called hydrosilylation to form a covalent bond between the dopant containing molecules and Si atoms of the substrate’s surface [70]. A conformal doped junction is formed by capping a thin SiO_2_ layer and high temperature RTP (rapid thermal processing) annealing to drive the dopant into the substrate.

The tuning of doping concentration in the MLD process could be realized by either changing the amount of the dopant atom in a molecule (dopant enriched adsorbate) to improve the packing density or by mixing the dopant adsorbate with molecules that lack dopants to reduce the dopant quantity at the interface. The final junction doping level is always determined by dopant solid solubility of the dopant element [71,74].

## 7. Plasma Doping

Plasma doping is a method based on when the dopants are adsorbed conformally on the surface of Si during the wafer, which is immersed into the plasma and dopant radicals in plasma. The uniform doping profile can be obtained by plasma doping and the damage to the surface can be suppressed by controlling the plasma energy. Recently, this technology has been improved by introducing ion energy decoupled plasma doping. This is based on a pretreatment to corporate decoupled plasma doping. In this way, the dopant level was remarkably raised and the surface damage could be decreased by heating the wafer during the process [68,75,76]. 

## 8. High-*k* & Metal Gate (HKMG)

In downscaling of MOSFET, SiO_2_ high-*k* material was eventually replaced with high-*k* material and the gate formation in the process flow was moved to the last in order to save the high-*k* integrity. 

Table 1 summarizes the thickness of the materials in the gate stack (high-*k* dielectric and SiO_x_) from a 45-nm to a 5-nm technology node [77,78,79,80]. The thickness of SiO_x_ decreased dramatically from ~1.2 nm in a 45-nm node to ~0.6 nm in a 14-nm node. In a similar way, high-*k* dielectric (HfO_2_) decreased from ~1.5 nm in a 45-nm node to ~1.2 nm in a 14-nm node. As a result, the equivalent oxide thickness of gate dielectric was decreased. In the 14-nm node CMOS, the thickness of SiO_x_ and HfO_2_ have significantly decreased to ~0.6 and ~1.2 nm, compared to ~1.1 and ~1.0 nm for transistors in a 22-nm node. The 0.6 nm of SiO_x_ contains only four or five layers of atoms, which is very thin. In addition, for reliability of the gate stack, the thickness of SiO_x_ is an important issue and, consequently, cannot be further decreased. Therefore, it is expected that the thicknesses of SiO_x_ and HfO_2_ would be considered ~0.5 nm and ~1.0 nm beyond a 5-nm node. 

Therefore, for high-*k* dielectric gate stack beyond the 5nm CMOS, almost the same gate stack (HfO_2_ and SiO_x_) will be built and the thickness of these two materials will be nearly unchanged compared with the 14-nm technology node. This is due to the direct tunneling current, which increases exponentially with thinner film thickness.

For the metal-gate, the N metal for NMOSFET (N-type metal-oxide-semiconductor field effect transistor) is still TiAl-based material, and the P metal for PMOSFET (P-type metal-oxide-semiconductor field effect transistor) is TiN. The work function metals for the NMOS (N-type metal-oxide-semiconductor) and PMOS (P-type metal-oxide-semiconductor) in 45 nm and 32 nm node were TiAlN and TiN, respectively [4,81]. Through the tremendous downscaling of CMOS starting from FinFET in a 22-nm node to the GAA (nanowire) structure, the electrostatic gate control is improved. This decreases the requirement of the metal-gate work function [41].

For the GAA structure, the gate-fill is a challenging task, and this increases the requirement of further decreasing the metal-gate thicknesses. Beyond the 5-nm technology node, the thicknesses of the TiAl and TiN metals are expected to be ~1.0 nm and ~1.2 nm, respectively.

For transistors beyond the 5-nm node, the device performance cannot be further improved by optimizing gate stacks. The high-*k* dielectric and metal-gate are simply very thin and cannot be further decreased. For the GAA device structure, the deposition of HKMG requires precision in atomic levels. The ALD technique offers a good control for layer thickness of HfO_2_ and TiN. However, for NMOSFET, it is relatively difficult to acquire the N-type work function metal due to the precursor limitation. In this field, TaC_y_ [82], TaCN [83], TiC [84], WC_0.4_ [85], and ErC_2_ [86] were studied for NMOSFET application. However, in most of these processes, plasma enhanced ALD (or PEALD) was used. To some extent, thermal ALD without plasma damage is more suitable for the metal gate. TiAlX films as the metal gate were developed by Chao et al. by the thermal ALD technique using TiCl_4_, TMA (Al(CH_3_)_3_), and NH_3_. It was demonstrated that NH_3_ presence in the TiCl_4_ and TMA reaction makes the film more like TiAlN(C) while its absence makes the film turn to TiAlC. The TiAlC film has a smaller effective work function than the TiAlN(C) film [87]. The effective work function can be tailored from 4.49 eV to 4.79 eV by tuning the process conditions [88]. By introducing the triethylaluminum (TEA) into the process, more Al-doping is obtained in the TiAlC film due the reaction of TEA with TiCl_4_. The effective work function of the TiAlC with TEA as a precursor can be tunable from 4.46 eV to 4.24 eV [89]. The deposition of TaAlC films using TMA and TEA has almost the same effective work function as TiAlC films grown by TMA and TEA separately, which provide more choices for metal gate selection [90,91]. The effective work function of different metals for NMOSFET are summarized in Table 2 [82,83,84,85,86,87,88,89,90,91].

High-*k* material can be applied for a more complicated transistor design, e.g., negative capacitance FET (NCFET). This type of transistor is a strong potential device beyond the 5-nm node CMOS. The reason for choosing NCFET is due to its dramatic improvement in a sub-threshold swing, which has compatible process flow with the conventional CMOS technology, and on-current enhancement [92]. The high-*k* materials suitable for NCFET are considered to be HfZrO and HfO_2_ with a thickness below 5 nm.

## 9. Interconnections in CMOS

Tungsten (W) has excellent thermal stability, the highest melting point among all metals, and perfect resistance to electro-migration (EM). The tungsten plug has been used for metal interconnection in integrated circuits to connect different layers of metals to nano transistors. ALD has been widely used for deposition of tungsten. ALD W has been used as gate filling metal (HKMG-last) due to its properties for trench filling [93]. ALD W can be selectively deposited and this is important for advanced sub-10 nanometer transistors, which needs good alignment to underlying structures, and edge definition [94]. W films have an α-phase and a β-phase with different morphologies and electrical properties. W films with an α-phase have the lowest resistivity and an important role in the logic MOSFETs. 

**Figure 14:**
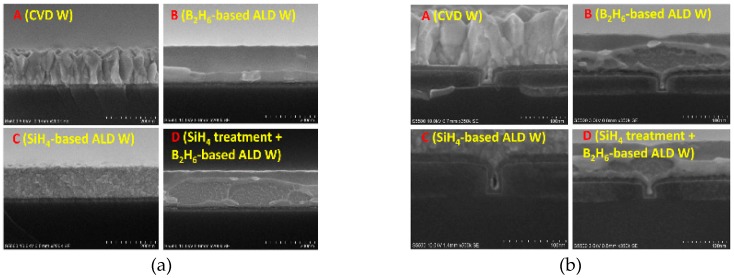
Cross-sectional SEM images of W films grown in different conditions: (**a**) on blank wafers and (**b**) trenches filling capacity [104].

The common precursors used for ALD W are SiH_4_ [95,96,97,98,99,100,101,102,103,104,105,106,107,108], Si_2_H_6_ [99], and B_2_H_6_ [100,101,102,103]. Different precursors will form films with different phases. Qiang Xu et al. studied the adhesion, roughness, and pore filling ability of ALD W films using different growth methods and different precursors for the 22-nm technology node, as shown in Figure 14 [104]. The authors demonstrated that the roughness of the W film grown by the ALD technique is clearly better than that of CVD. The roughness of ALD films grown by SiH_4_ is better than that of the B_2_H_6_ precursors. However, the filling performance of SiH_4_ is worse than that of B_2_H_6_.

**Figure 15:**
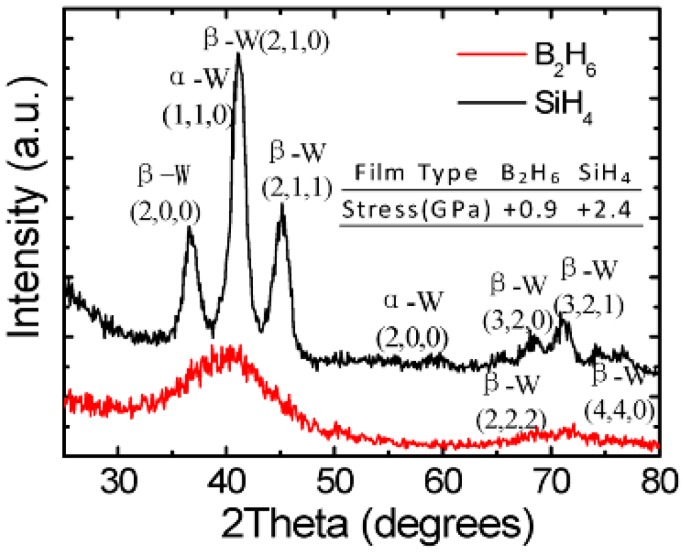
XRD spectra of ALD W using SiH_4_ and B_2_H_6_ and calculated stress data on a blank substrate [98].

In order to further understand the internal morphology of the ALD W film, Wang et al. measured the morphology of these films by using the XRD technique. It was found that there were two kinds of crystalline phases in the ALD W films grown by SiH_4_ as precursors, while the films grown with B_2_H_6_ were amorphous, as shown in Figure 15 [98].

In integrated circuits, ALD W with the α-phase is commonly used for metal interconnection or electrode filling. One way to grow tungsten films with α-phase on SiO_2_ is to use WF_6_ as a precursor and H using hot-wire (HW) assisted atomic layer deposition (HWALD) [105]. Kim et al. demonstrated that tungsten films using B_2_H_6_ and WF_6_ precursors create large grain size α-phase tungsten at 450 °C. Meanwhile, at 395 °C and applying a low flow rate of B_2_H_6_, smaller grains could be obtained [106].

The initial nucleation process in the growth of ALD has a great influence on the state of the subsequent films. The main factor affecting the nucleation of ALD is the surface active site density. For example, in the study of selective ALD deposition, it was found that hydroxyl bonds were formed on the surface of SiO_2_ after wet cleaning, which resulted in the nucleation of SiH_4_ and WF_6_ on the surface of SiO_2_ [94]. However, the nucleation is greatly delayed after the removal of surface hydroxyl groups by heating or using a precursor, which interact with the surface to change the surface characteristics. For example, when B_2_H_6_ and WF_6_ are used as precursors, pretreatment with B_2_H_6_ accelerates nucleation. At the same time, the resistivity of the film is reduced.

F.H. Fabreguette presented the Quartz crystal microbalance study of the tungsten atomic layer deposition using WF_6_ and Si_2_H_6_. This work found that the growth rate of ALD W was weakly temperature-dependent with an activation energy of 1.5 ±0.1 kcal/mol at T < 250 °C and a lower activation energy of 0.6 ± 0.3 kcal/mol at T > 275 °C [99].

At present, there are few research studies on the theory and interfacial states of ALD W. It is a valuable research direction to study the selective deposition on the basis of interfacial states and the formation mechanism for different crystal phases of W in the future. In the aspect of the device application, the process of preventing the diffusion of the F atom and the B atom through the TiN/Ti layer is also a valuable research direction in the integrated circuit.

## 10. Stressors SiN_x_ Contact Etch Stop Layer (CESL) Technology

After integration of the SiGe stressor material in MOSFETs, a large effort was spent to find new methods to increase the strain amount. Among the various methods, stress liner technology, which is based on the SiN_x_ contact etch stop layer (CESL) received more attention. The strain in these films could be tuned from highly tensile to highly compressive. The stressed nitride contact liners were incorporated into a high performance CMOS flow. This CESL approach resulted in N-FET/P-FET effective drive current enhancement of 15%/32% and saturated drive current enhancement of 11%/20%. In these transistors, a significant enhancement of 60% was achieved in whole mobility without using SiGe [107].

**Figure 16:**
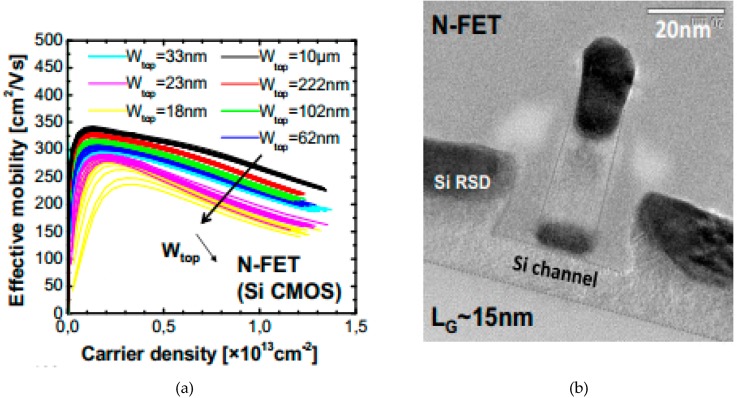
(**a**) Electron effective mobility in NFET and (**b**) TEM images Ω-Gate CMOS NW transistors for N-FET [108].

Another example is shown in Figure 16a,b where the CESL was used in Ω-gate CMOS NWs with N-FET. The carrier mobility of transistors lied in a range of 250 to 350 cm^2^/Vs for different gate widths [108].

### 10.1. High Tensile Stress CESL

Many growth techniques have been applied to deposit high tensile SiN_x_, e.g., LPCVD (low pressure chemical vapor deposition), ALD, and PECVD (plasma enhanced chemical vapor deposition). It is known that Si_3_N_4_ films produced by the LPCVD technique possess high tensile stress of 2 Gpa. However, the relatively high thermal process makes the process not compatible with Ni silicide [109]. Therefore, PECVD technology with a low thermal budget process was taken as the best choice to deposit CESL [110]. 

**Figure 17:**
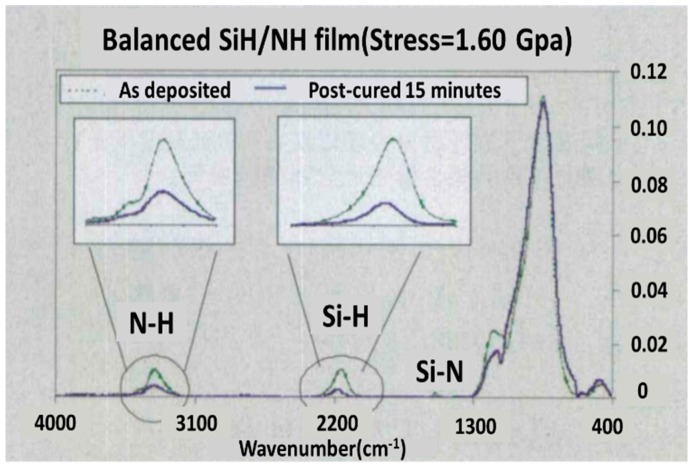
Tensile stress change with a UV cure.

Unfortunately, the hydrogen in PECVD nitride film could not have been pushed out, which pulled down the film tensile stress to about 1 Gpa [111]. There are various reports that demonstrate methods. Plasma treatment and the ultraviolet thermal process (UVTP) can enhance the tensile stress. The latter method breaks Si-H and N-H bonds and pushes out H molecules. This method meets the demand of both high tensile stress, a low thermal budget, and stress amount as high as 1.7 Gpa can be obtained (see Figure 17).

### 10.2. High Compressive Stress CESL

In order to make high compressive stress CESL, both the RF power source and diluted gas have to be tuned. It is known that the compressive stress has a strong relationship with which a type of diluted gas is used. For example, the compressive value is as low as ~1.2 Gpa when nitrogen is used as diluted gas whereas a mixture of argon and nitrogen could highly increase the compressive stress to ~2.3 Gpa [112]. The compressive strain could be further increased to ~3.1 Gpa by using a diluted gas of hydrogen and argon mixture. The hydrogen reduces the energy loss during bombardment. To obtain further improvement, it is necessary to improve the film’s elasticity modulus by applying the carbon element, which could impel hydrogen volatilize (less hydrogen and higher compressive stress). In this case, the SiH_4_ precursor has to be replaced with TMS (tetramethylsilane), which contain a carbon element and compressive stress could reach close to 3.5 Gpa.

## 11. Etching Evolution 

Miniaturizing the transistor according to the principles shown in Figure 2 occurred when equivalent oxide thickness (EOT), transistor gate length, and transistor width were scaled down by a constant factor. However, this trend is followed very differently when the CMOS scaling focuses more on low voltages and low power consumption. By entering the 10-nm technology node, the silicon channel is being gradually replaced with silicon-germanium (SiGe), germanium (Ge), or III-V materials because they have remarkably higher carrier mobility [113]. For example, 40,000 cm^2^·V^−1^·s^−1^ for InGaAs [114] (for electrons) and 1900 cm^2^·V^−1^·s^−1^ for Ge [115] (for holes) compared to 1400 cm^2^·V^−1^·s^−1^ for electrons and 450 cm^2^·V^−1^·s^−1^ for holes in silicon [116]. Not only is the channel material changed, but the transistor shape is changed from a simple fin-like shape to a lateral gate all around (LGAA) or vertical gate all around (VGAA) in order to obtain transport control through the channel region. It means that the fabrication of state-of-art transistors need to be modified. 

In this case, a dummy gate poly crystalline was initially formed as a replacement metal gate (RMG) as well as silicon dioxide, which was deposited as dummy gate oxide to give the green light for all the high temperature-annealing processes [117]. Eventually, the gate was removed by a wet process using none metal alkaline solutions. The merits of RMG are first addressed to avoid crystallizations of the high-*k* dielectric during the rapid thermal annealing (RTA) process for dopants activation, which may increase leakage current of the gates [117]. Second, it avoids the chemical reactions between the metal-gate and the high-*k* in RTA processes [118] or it avoids the boron diffusion into high-*k* [118,119].

**Figure 18:**
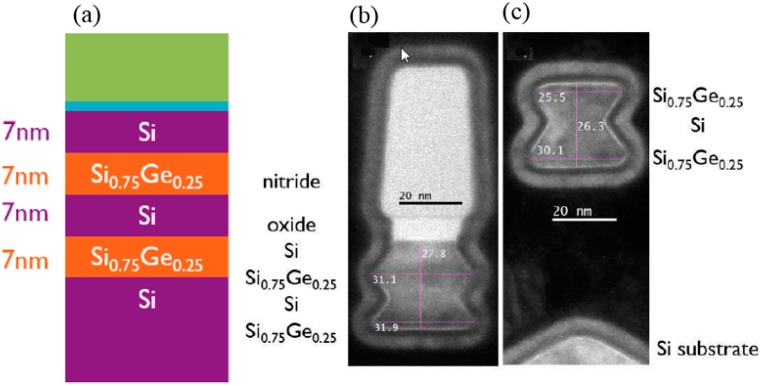
(**a**) Schematic picture of a stack of Si_0.75_Ge_0.25_/Si NW used for selective etch and TEM across a section of 30-nm wide Si-Si_0.75_Ge_0.25_ NWs after Si selectively etched (**b**) in TMAH 5% and (**c**) without the oxide-nitride HM [127].

The RMG process is still used and will be applied for the 7 nm and 5 nm technology node, where the sacrificial material is Si and will be selectively removed from the SiGe channel [120]. The exponential decrease in the alkaline etch rate of SiGe with increasing Ge content enables the selective removal of Si to Si_0.75_Ge_0.25_. [121,122,123,124]. Alkaline etching of Si has been extensively studied and is well understood [125,126]. The etch rate in Si (001) and (110) directions is remarkably faster compared to the Si (111) crystallographic planes [127]. As shown in Figure 18, the selective etching of Si to SiGe was performed at a TMAH 5% solution at 60 °C. In both pictures, the Si underneath material of the NW stack is completely removed. The 7-nm thick Si layers, which are sandwiched in between the Si_0.75_Ge_0.25_NWs are removed until the (111) limiting planes are formed.

In fabricating the SiGe NWs from the Si/SiGe stack, the conventional alkaline Si etchant such as TMAH (aq) poorly removes the Si sacrificial layer. The selectivity of the Si-vs-SiGe etch is only marginal [128,129]. A surface modifier is employed in ACT^®^ SG-201, which improves the relative etch rates of Si (110) and Si (111) orientations. This solution results in etching selectivity of Si (110)/Si (100) in the range of 1 to 2.5 and Si (111)/Si (100) of about 0.5 or above. By a combination of the Si surface modifier and an effective SiGe corrosion inhibitor in ACT^®^ SG-201, the selectivity of Si (111)/SiGe 25% is significantly improved as compared to the conventional Si etchants. Consequently, ACT^®^ SG-201 is more efficient in removing the sacrificial Si layer in the Si/SiGe stack [130]. The reduced Si etch rate anisotropy in combination with an effective SiGe corrosion inhibitor prevents SiGe loss during the NW release [130].

**Figure 19:**
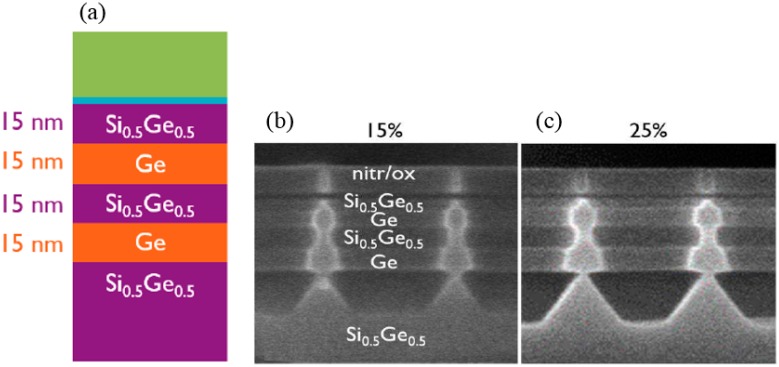
(**a**) Pictorial description of the Si_0.5_Ge_0.5_/Ge NW stacks used for the selective etch test and SEM images of Si_0.5_Ge_0.5_-Ge NWs after selective etch for 10 seconds in TMAH with a concentration of (**b**) 15% and (**c**) 25% for 45 and 55 nm wide fins [127].

The sacrificial material is SiGe and will be selectively removed from the Ge channel [131,132,133]. For example, SiGe can be selectively etched to Ge in diluted TMAH 5–25% at 90 °C. Figure 19 shows the Si_0.5_Ge_0.5_/Ge NWs after selective etching in TMAH 15 or 25%. The Ge NW is not etched in the solution while the Si_0.5_Ge_0.5_ and substrate are etched anisotropically. The undercut for the 25% TMAH solution is more than for the 15% solution one. The etch rate of the (001), (110), and (111) crystallographic planes of Si_0.5_Ge_0.5_ were estimated from these cross-sectional scanning electron microscopy (XSEM) images (see Figure 19b,c). The etch rate of the different planes is decreasing in magnitude from (001) to (111) to (110) plane. If the selective removal of Ge is the goal, then oxidizing solutions, e.g., using SC1 solutions can be used due to the high solubility of the Ge-oxide [122,134].

In fabricating the Ge NW from the SiGe/ Ge stack, two different (i.e., alkaline vs acidic) chemical solutions have been investigated. The alkaline solution (5% TMAH) shows anisotropic SiGe etch behavior as well as a decrease of etch rate when the Ge content is higher than 50%. To overcome the anisotropic etch problem in the alkaline solution, isotropic SiGe etch behavior and better Ge protection are required. Different from the Ge etchant with mixtures of HF-H_2_O_2_-H_2_O [135], the formulated solution ACT^®^ SG-301 employs a selective oxidizer [130], a SiGe etchant, an effective Ge corrosion inhibitor, and a well-designed solvent system for polarity adjustment. By suitable pH control, the oxidized SiGe sacrificial layer could be effectively removed and the Ge NW damage could be minimized [130].

## 12. BEOL for Nano-Scale Transistors

Interconnects are a fundamental element of any microelectronic circuit. As semiconductor technology keeps evolving along the trajectory predicted by the Moore’s Law, the CD of the BEOL circuits must continue shrinking. However, the scaling of the interconnect dimensions will be led to the deterioration of the interconnect performance and reliability [136]. Twenty years ago, dual damascene copper replaced the subtractive etch of aluminum and the method for BEOL interconnect fabrication [137]. Copper is expected to be used in scaled transistors in the future. However, as the CD narrows, filling the BEOL trench over the structure using the conventional physical vapor deposition-Electro chemical deposition (PVD-ECD) approach becomes more and more challenging. In the 5-nm technology node, interconnect half pitches are expected to reach dimensions of 12 nm [138,139]. For such narrow lines, the intrinsic properties of Cu start to severely limit the interconnect performance. At first, Cu resistivity is increased because of electron scattering at the sidewall and grain boundaries [140,141,142], which results in an exponential increase in resistivity and resistance. Secondly, there are limitations in scaling the diffusion barrier for the currently used Cu dual-damascene process, which increasingly reduces the Cu volume in interconnect lines [143,144]. Thirdly, barriers and liners do not scale well since strongly reduced thicknesses negatively affect the dielectric breakdown as well as the electromigration (EM) properties [144]. Hence, these issues eventually stop scalable solutions for interconnects. 

Materials innovation and integration improvement are the requirements for diffusion barriers in combination with low-*k* dielectrics, the resistance to EM, and lower resistance than the combination of Cu and barrier layers in small dimensions. According to these requirements, there are two methods to improve them. One is to partially modify traditional Cu integration process and the other is to use new materials to replace the Cu integration process.

As the critical dimension continues to shrink to a 5-nm node, the bilayer approach (PVD TaN/Ta) faces scaling challenges, e.g., thickness control and PVD TaN over-hang. Wu et al. reported a novel approach to use thin (≤ 15 Å) ALD-based TaN barriers [145] and Co liner instead of copper electroplating. The use of a post-ALD treatment in a PVD chamber resulted in ALD films with resistivity, density, and a Ta/N ratio similar to industry-standard PVD TaN. This approach enables the conformal Cu barrier without reliability degradation compared to PVD TaN [145]. This new method overcomes the shadowing effect of the traditional PVD approach and improves the metal-fill process window. Furthermore, this method promotes lower via-resistance through barrier thickness reduction, which proves it to be a viable Cu-barrier candidate for the 5-nm node and beyond. However, this approach would only be a short-term alternative due to a size effect of Cu resistivity and TaN high resistance [146].

**Figure 20:**
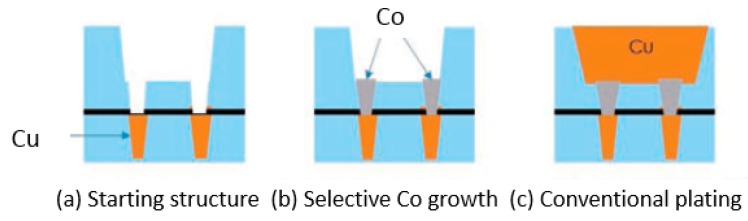
Integration of Co selective growth [148].

Van der Veen et al. [147] and Zhang et al. [148] introduced Co via-prefill concept to achieve void-free and bottom-up fill of metal in advanced interconnects, as shown in Figure 20. The via-prefill is beneficial for the Cu damascene process. The direct contact of Co and Cu at the bottom without TaN/Ta barrier interface results in a reduction of via-resistance. Moreover, Co is expected to have a better EM performance compared to Cu due to its higher melting point [143,146,149,150].

Zhang et al. reported, for the first time, a highly selective CVD Co deposition on Cu to fill a 45 nm diameter with 3:1 aspect ratio in a Cu dual damascene structure [148]. The results showed void- free Co-fill of the vias. interuniversity microelectronics centre (IMEC) demonstrated the feasibility of the prefill concept using the electroless deposition (ELD) technique for Co as material to pre-fill vias [147,151,152]. The main benefit of having Co vias is the reduction of the via-resistance. As the via CD shrinks, vias with ELD-Co show larger resistance reduction compared to the conventional PVD-EC Dones. As an example, at 40 nm via critical dimension (CD), the via-resistance reduction is ~30% [151]. For 12 nm chamfered vias with 3 nm metal barrier and Co via prefill, 45% resistance reduction can be achieved [152]. Therefore, the selective Co process for contact and via prefill has the potential to enable future scaling of the advanced technology node. 

**Figure 21:**
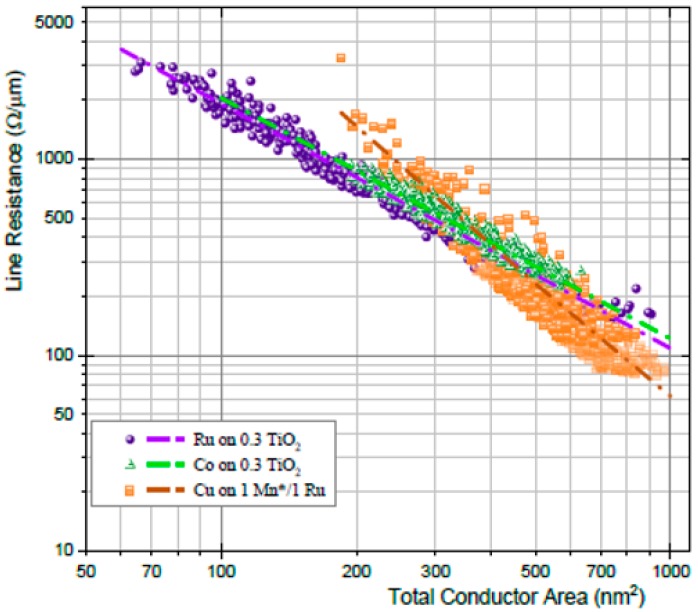
Damascence line resistance vs. the total conductor area of Ru, Co, and Cu NWs [142].

Marleen et al. benchmarked Ru, Co, and Cu in a damascene vehicle with scaled dimensions down to 11 nm CD and for different aspect ratios [142]. The Ru and Co NWs have higher resistivity, but Ru and Co are both superior to Cu for trenches smaller than 250 nm^2^. The difference in resistance for the Ru, Co, and Cu is clearly increased with the decrease of the total wire area, as illustrated in Figure 21 [142]. The slope for Cu increases and it crosses over to Co and Ru at 400 nm^2^. Ru effective resistance potentially crosses the Cu curve at 14 nm by assuming that the Cu barrier/liner thickness was 2 nm. The cross-point would happen at 8 nm with 1 nm Cu barrier/liner thickness [141]. Moreover, the EM performance reveals that the barrier-less Ru systems are robust with higher lifetime compared to Cu and Co [142]. These properties make Ru an attractive interconnect candidate for small line widths.

ALD Ru was studied as an option for barrier-less metallization for the future interconnects [141]. Ru shows regular nucleation on SiO_2_ without any growth inhibition. The adhesion was significantly increased to 7.0 ± 2.3 J/m^2^ by applying an ALD TiN adhesion promoting layer with a thickness as low as 0.25 nm. The Ru lines with widths of about 10 nm, which show excellent EM behavior on a single damascene test vehicle. Time-dependent dielectric breakdown measurements revealed negligible Ru ion drift into dense low-k dielectrics with k ∼3.0 up to 200 °C, which demonstrates that Ru has the potential to be used as a barrierless metallization as a future advanced interconnect solution.

Currently, the damascene implementation of Ru lines is hampered by the availability of optimized CMP. A semi-damascene integration approach is a proposed solution for the multi-level Ru interconnect [153]. This method is formed in low-k and then followed by filling both the via and the trench layers with a single deposition step. Key advantages are that the process can be barrier-less, the grain size can be tuned, and there is no requirement for metal CMP. The trench layer is then patterned using subtractive etch, which eliminates the need for plasma processing of low-*k* trenches. Ru films were patterned using EUV single exposure and subtractive etch to generate lines with CD down to 10.5 nm. This approach has excellent process control, stability, and results in a very high line yield. These results indicate that the subtractive etch of Ru could be a viable interconnect candidate for advanced technology nodes.

**Figure 22:**
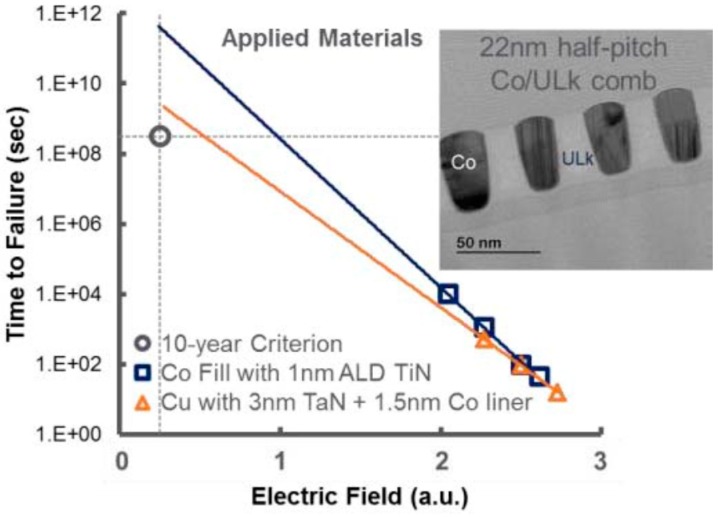
22-nm half-pitch Co lines/ALD TiN liner compared to Cu [139].

**Figure 23:**
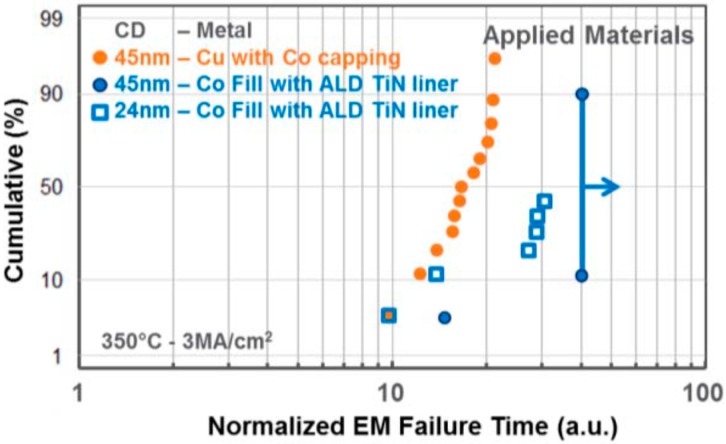
Performance of Co and Cu EM with 1-nm ALD TiN liner [139].

Plasma enhanced chemical vapor deposition Co was evaluated to fill dual damascene (DD) structures as an interconnect wiring metal alternative to Cu [139]. The void-free gap fill of damascene structures down to 10 nm CD was demonstrated using just 1 nm ALD TiN liner. A CMP process without Co residues or corrosion has been developed. 22 nm half-pitch Co lines with 1 nm ALD TiN liner in porous ultra low-*k* (ULk) meet the 10-year lifetime time-dependent dielectric breakdown (TDDB) reliability requirement in Figure 22 [139]. EM data indicates that the Co EM performance with 1 nm ALD TiN liner can be better than that of Cu in Figure 23 [139].

**Figure 24:**
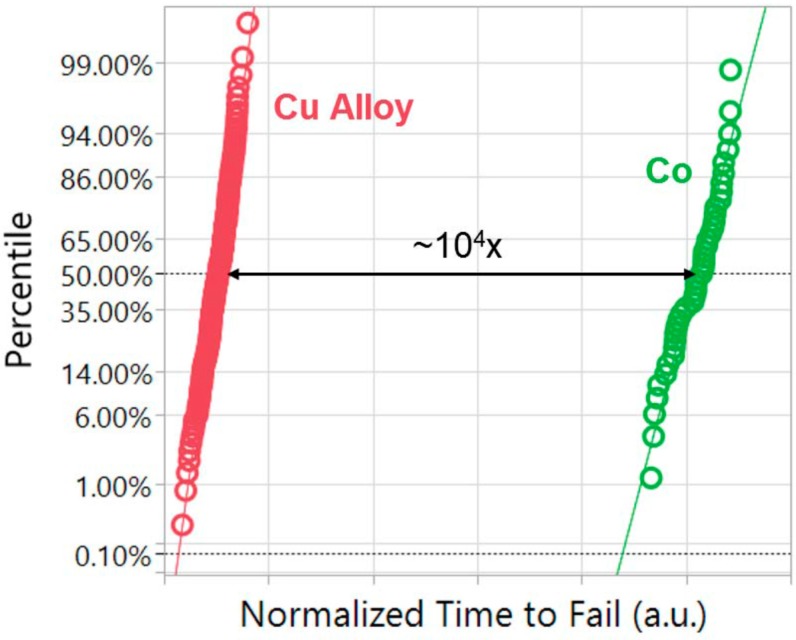
EM lifetime distributions for the Co and Cu alloy [157].

In response to the scaling challenges, Intel introduces a new metallization solution to meet the reliability challenges of technology scaling [154]. At trench contact, electroplating of Co occurs on a chemical vapor deposition (CVD) TiN barrier/adhesion and CVD Co seed layer. EM time to failure is observed to be at least four orders of magnitude higher for Co fill interconnects compared to the Cu alloy (see Figure 24). Moreover, superior intrinsic TDDB and stress induced voiding reliability was also demonstrated for Co low-*k* interconnects. Co shows superior intrinsic properties with respect to Cu.

Chemical vapor deposited tungsten (CVD W) based middle-of-the-line (MOL) contacts and local interconnects have been extensively used in high-performance CMOS logic IC’s. The standard process scheme has included a TiN adhesion layer to dielectrics and a nucleation W layer for CVD W, but these layers consume most of the volume in narrow features. A major challenge for W fill scaling is that the line resistance of sheet Rs and plug resistance of contact Rc increase due to a reduction in the volume of the low resistance CVD W bulk material. Recent simulation results of the local interconnect resistance indicated that the M0 and the contact would become dominant contributors to a resistance increase at the 5-nm process node [155]. W M0 and Contact comprise 65% of the total M0/M1 stack resistance due to a narrow CD. Contact shows 43% reduction in M0/M1 resistance.

CVD Cobalt (Co) has been used in recent studies as replacement for W in local interconnect showing a 2.5× line resistance reduction, void-free and seamless fill at the local interconnect level [156]. Unlike W, Co metal does not need high resistivity nucleation layers and can be annealed at low temperature to undergo grain growth and reflow into the high aspect ratio contact plug. Moreover, CVD Co precursors do not attack Ti liner, which enables barrier thickness scaling. The resistivity of Ru [157] is comparable to that of Co in terms of 7 nm MOL critical dimensions. Both Ru and Co have better liner/barrier scalability compared to W. Susan Su-Chen Fanet al. demonstrated Ru metallization assessment on 7 nm MOL with remarkable resistance reduction in the S/D contact and MOL local interconnect [158]. Table 3 summarizes some MOL Metallization Options [158].

Moreover, the Co/CoTi_x_ structure on SiO_2_/p-Si was investigated to evaluate its feasibility to replace conventional W/TiN/Ti structure of MOL in a future technology node [158,159,160]. An alloy of Co-20 at.%Ti is chosen as a single layer liner/barrier to replace Ti/TiN. Since the binary phase diagram of the Co-Ti system shows a deep eutectic point at 24.2 at.%Ti, an amorphous phase can be formed as a metastable state, which is a prerequisite structure for a barrier layer. Good adhesion and low film resistivity between Co and SiO_2_was attained by CoTi_x_ in as-deposited and annealed samples. The results indicate that the 3 nm thick CoTi_x_ layer has an excellent diffusion barrier property against Co diffusion at the elevated temperature 500 °C. Therefore, the amorphous CoTi_x_ alloy could be a promising liner/barrier material in combination with the Co M0/contact structure for advanced technology of the 5-nm node.

## 13. Reliability

For advanced CMOS technology, the ultra-scale FinFET and Nano sheets are the best candidates for the beyond 5-nm technology node. The reliability issues are becoming more complicated due to the novel materials, novel process, novel integration, and novel structures for the performance improvement. 

### 13.1. New Material—Ge/GeSi

**Figure 25:**
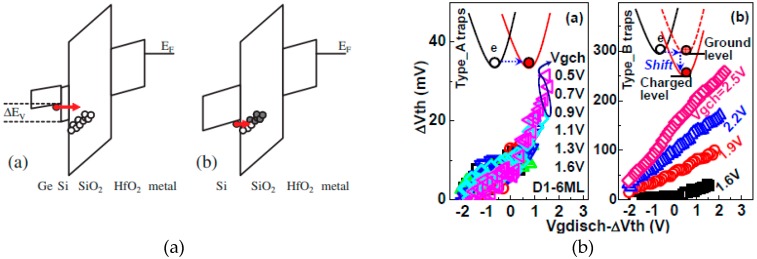
(**a**) Schematic image of the band alignment of the Ge and Si channel gate stacks during NBTI stress. The Ge/Si valence band offset (Si-passivated Ge FETs) causes the inversion layer to energetically move away from the oxide traps [161] and (**b**) defect energy profiles after filling at low and high Vgch vs. Vgdisch-ΔVth. The insets illustrate the charging mechanisms for two different types of electron traps [162].

In order to improve the device performance, the novel materials, such as GeSi/Ge, is applied in channel or S/D regions [161,162,163,164,165]. In contrary to traditional silicon technology, the structure of high-*k* and metal gate (HK & MG) stack is changed/improved by novel material application but, at the same time, the interface quality between substrate and gate insulator becomes worse. Therefore, interface quality and reliability of novel materials are actively researched. Based on those, a Si-passivated option was proposed by inserting a thin silicon passivated monolayer between Ge layer and SiO_2_ to improve interface quality and reliability together [161,162,163]. In this case, the band alignment of Ge and Si shows the different carrier transport mechanism under bias temperature instability (BTI) stress. Therefore, the BTI is clearly improved, as shown in Figure 25a. Moreover, in Figure 25b, the two types of electron traps are investigated, which is related to the HK layer thickness and Si-cap growth condition [165]. Furthermore, this is a big issue for the reliability model for new materials, such as the negative bias instability (NBTI) model of SiGe [166,167]. The novel materials can worsen the interface quality and cause serious reliability problems. Therefore, the solution to suppress reliability degradation and to improve performance are important issues.

### 13.2. New Process—Dipole Formation

**Figure 26:**
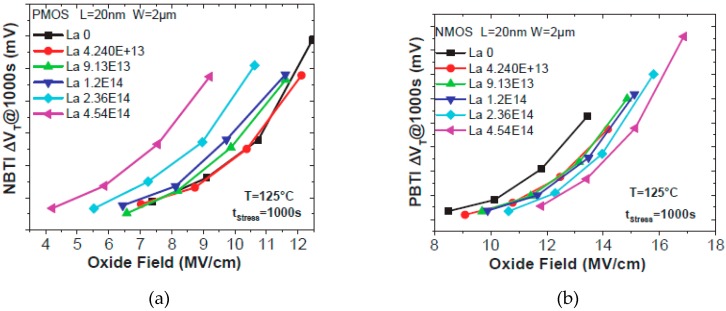
(**a**) NBTI V_T_ shift vs. Eox after 1000 s for all effective La doses in PMOS. La addition causes enhancement of NBTI V_T_ degradation and (**b**) PBTI V_T_ shift vs. Eox after 1000 s for all La doses for NMOS. La addition causes reduction of PBTI V_T_ degradation [168].

In advanced CMOS technology, in order to reduce the influence of the thermal budget on junction and channel quality, introducing the novel materials and processes are necessary in gate engineering. Therefore, the dipole formation in the gate stack is widely applied in the Replacement Metal Gate (RMG) process [168,169,170,171,172,173]. Usually, Lanthanum (La) and Aluminum (Al) are used to tune threshold voltage for NFET and PFET due to the different dipole polarity [168,169,170,171]. This includes metal deposition (La or Al) and annealing treatment with or without a capping layer [168]. It has been reported that La induced dipole effectively, which makes V_T_ decrease and positive bias temperature instability (PBTI) improve, but NBTI is worsened [168,169]. Meanwhile, the Al induced dipole increases V_T_ and BTI becomes worse as well. These results are explained by the band-diagram at the same E-field and Vg stress, as shown in Figure 26 [168]. Recently, IBM and the Applied Material proposed ALD Mg-based dipole method for multi-V_T_ tuning application. The results discuss the electrical parameters changing by the Mg-based dipole. However, there are no reliability investigations [168].

### 13.3. New Integration—Physical Mechanism

**Figure 27:**
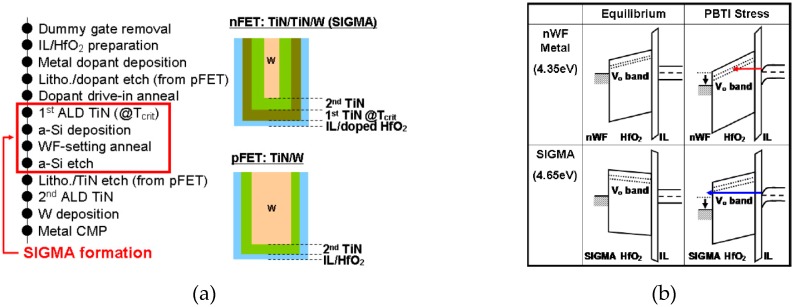
(**a**) CMOS flow and schematics for NFET with SIGMA/W stack and PFET with TiN/W stack and (**b**) PBTI improvement mechanism for the SIGMA stack [174].

Similar to the novel process, the novel integration is also necessary for performance and reliability improvement especially in gate engineering [162,163,164,174,175]. As an example, Simple Gate Metal Anneal (SIGMA) [171] is applied for a thin TiN layer as a work function (WF) metal for CMOS (red-marked in Figure 27a). The PBTI lifetime is 100× improved due to the oxygen vacancy effectively decreasing during α-si removal. The CMOS integration flow together with a mechanism for PBTI improvement, which are shown as a band diagram in Figure 27b. For the reliability study, the impurity implantation in the HK&MG stack, such as “Nitrogen Implantation” in the work function metal layer [170] and the thickness tuning of an effective work function metal (EWF) can be investigated [170,171,172,173]. The advantage of such a study is to control the V_T_ shift over a processed wafer, which provides very valuable information for chipmakers. This is a feedback between reliability improvement and novel integration.

### 13.4. New Structure—Self-Heating (SH) and Random Telegraph Noise (RTN)

**Figure 28:**
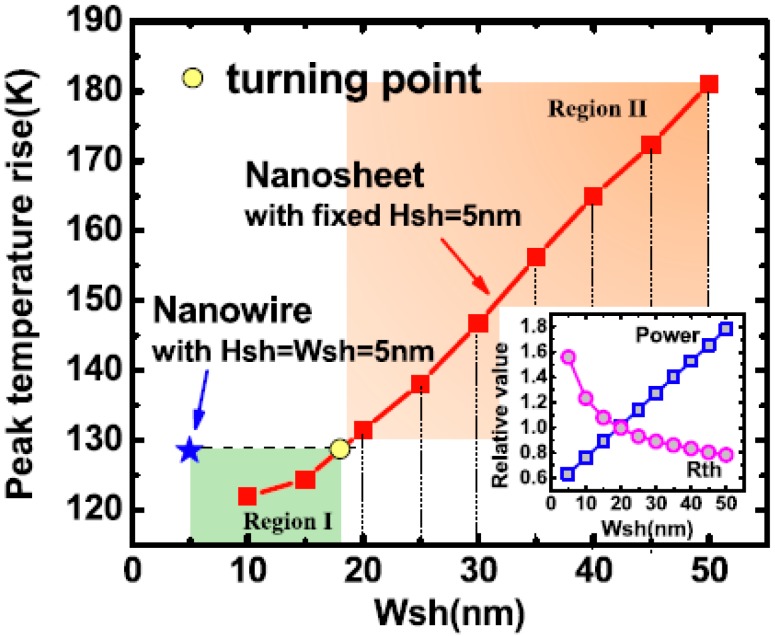
Peak temperature rise in nano-sheet FETs with increased width of nano-sheets (Wsh) [179]. H_sh_ stands for height or thickness of nano-sheets.

**Figure 29:**
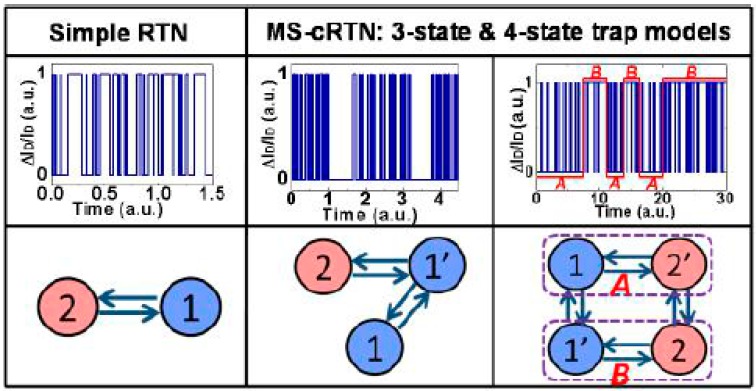
Illustration of the complex RTN induced by a single trap with an additional one or two metastable states, named as MS-cRTN [181].

In CMOS miniaturization, the random telegraph noise (RTN) needs to be paid more attention to as an indicator for problem source acting on the transistor performance. For advanced CMOS, the self-heating becomes a more serious matter and this has been widely studied [176,177,178,179]. It has been reported that PFET has higher RTN than NFET due to an extrinsic origin caused by SiGe in the S/D [177]. Moreover, based on simulation results, the nano-sheet devices exhibit better resilience to a self-heating effect (SHE) in comparison to the FinFETs [177]. In general, SHE is very sensitive to layout design, hot-carrier degradation (HCD), and bias temperature instability (BTI) [178,179]. In the layout design of the nano sheet devices, the width of the nano sheet (Wsh) is the key parameter, which provides a flexible choice to make trade-offs between thermal properties and electrical performance in nanosheet FETs, compared with the NW FETs (see Figure 28) [179]. Usually, the random telegraph noise (RTN) is generated by a single trap, which is explained by the “normal” two-state trap model. This model considers the RTNs caused by two or more traps in a device, which are regarded as independent and may have a superposition effect [180,181,182,183]. In general, two categories of RTNs are induced: those by the metastable trap-states and the others by the trap coupling effect, as shown in Figure 29 [180,181]. It has proposed a novel RTN-true random number generators (TRNGs) design, which provides a solution to address speed, design area, power consumption, reliability, and cost simultaneously [182]. In the RTN study of Ge NW nMOSFETs, it shows that the low frequency noise decreases when the channel length scales down from 80 nm to 40 nm because of the near-ballistic transport of electrons [183].

## 14. Channel Materials for Beyond Moore Era

### 14.1. III-V on Silicon

Chipmakers have declined III-V materials at the 7-nm node but the main question still remains whether III-V will happen in advanced CMOS. 

There are a lot of challenges in integrating III-V materials on silicon, ranging from epitaxy to etching. For example, the etch rates for ternary compound materials, e.g., InGaAs with complex concentration has to be properly controlled where all atoms will be removed at the same pace. Otherwise, the diffusion of arsenic during a different process could lead to cross-contamination [36].

Whatever, the next step in CMOS technology is, we have to implement the right process for integration of III-V materials on silicon, which are categorized in the three following ways: blanket epitaxy [184,185], selective epitaxy [186,187], and wafer bonding [188].

**Figure 30:**
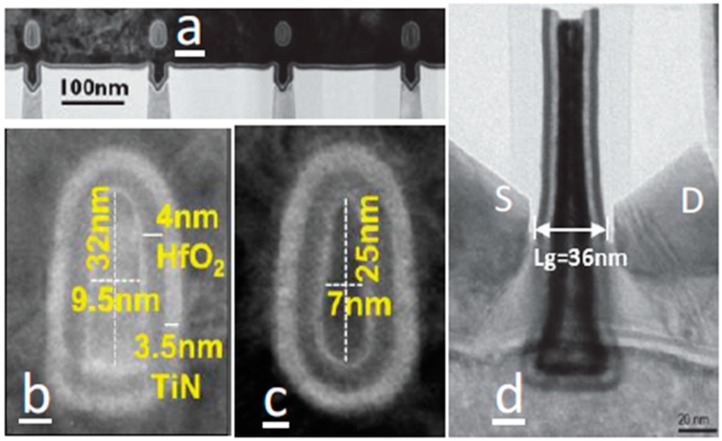
TEM micrographs of completed devices. (**a**) Devices across one gate pattern. (**b**) 9.5 nm-wide channel obtained from 2 cycles of WHALE* where 1 nm conformal interfacial layer was grown by ALD before HfO_2_ deposition. (**c**) 7 nm-wide channel obtained from 5 cycles of WHALE* with the same gate stack as shown in (**b**) are used, and (**d**) along the trench showing *L_g_* of 36 nm [186]. * WHALE stands for Wet HCl-based Atomic Layer Etch.

IMEC demonstrated III-V FinFET and III-V parallel Gate-All-Around (GAA) FET on a silicon substrate by ART technology [186]. They reported InGaAs GAA FETs with channel width down to 7 nm and *L_g_* down to 36 nm, which is the smallest dimensions reported about III-V materials devices on 300 mm Si wafer. Figure 30 shows the TEM of completed devices. I_ON_ above 200 μA/μm is obtained at I_OFF_ of 100 nA/μm and V_DS_ = 0.5 V on a 300 mm Si platform. The InGaAs S/D improves the peak g_m_ by 25% relative to InAs S/D.

The same group presented a promising way to manufacture III-V vertical GAA FET [187]. They used an InGaAs nucleation layer for InAs NW SAE on Si and reached nearly 100% yield, but there was no further report on the III-V vertical device yet.

In recent years, IBM demonstrated a series of electronic devices including InGaAs-OI FinFET, InGaAs FET, III-V Tunnel FETs (TFETs), and hybrid InGaAs/SiGe CMOS on a silicon substrate [189,190,191,192,193,194] by using advanced template-assisted selective epitaxy technology [190]. 

**Figure 31:**
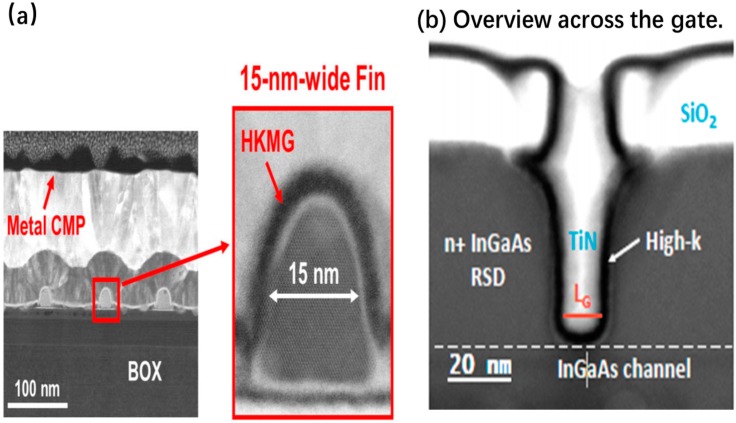
Cross-section images of the self-aligned InGaAs-OI FinFET architecture where (**a**) the scaled HKMG deposited on a 15-nm-wide fin using a highly conformal and uniform PEALD* process [192], and (**b**) shows CS STEM images across the gate showing the InGaAs FinFET with *L_g_* = 13 nm [194]. * plasma-enhanced atomic layer deposition.

They reported CMOS-compatible n-channel InGaAs-OI FinFETs by replacing the metal gate (RMG) process flow, as shown in Figure 31 [191,192,193]. The channel down to *L_g_* = 50 nm with W_Fin_ = 15 nm has proven to have an excellent control on short channel effects [192]. The same device exhibited a record I_ON_ of 156µA/µmfor a supply voltage of 0.5 V and a fixed OFF-state current of 100 nA/µm, with a minimum sub-threshold swing of 92 mV/decade at V_DS_ = 0.5 V and a drain-induced barrier lowering to 57 mV/V. This I_ON_ value is the highest reported to date for CMOS-compatible InGaAs devices integrated on Si. 

Furthermore, they show record-performance InGaAs-on-Insulator FinFET with *L_g_* down to 13 nm, where I_ON_ reaches to 249 µA/µm at fixed I_OFF_ = 100 nA/μm and V_D_ = 0.5 V. This work demonstrates the feasibility of high-performance III-V devices on Si at sub-7 nm nodes [193].

### 14.2. 2D Channel Materials

Over the last decade, various 2D materials have been discovered and studied as promising candidates for next-generation electronic materials due to their unique properties such as atomic thin thickness, high mechanical strength, transparency, and flexibility. There are several interesting 2D materials, which could be applied as channel material in CMOS for beyond the Moore era, as presented below.

#### 14.2.1. Graphene

Graphene is a material composed of carbon atoms closely packed into a single layer two-dimensional honeycomb lattice structure. Graphene has many unique physical properties, which attract attention to use it as channel material beyond Moore.

(1) Ultra-high mobility: The mobility of the suspended exfoliated graphene is as high as about 2 × 10^5^ cm^2^·V^−1^·s^−1^ without considering the charged impurities and ripples [195]. (2) Great thermal conductivity: The thermal conductivity of graphene is superior to that of carbon nanotubes and diamonds. Single-layer graphene has a thermal conductivity of up to 5300 W/mK, which is much higher than metals such as silver and copper with high thermal conductivity [196]. (3) Excellent light transmission: Graphene is almost completely transparent. It only absorbs 2.3% of light and allows all spectral light to pass evenly. Therefore, it also has great application potential in the field of optoelectronic devices [197].

Since the material has almost no bandgap, then a lot of works have been performed to create bandgap for graphene, which can be classified into three types: (1) fabricate graphene nanoribbons (GNR) by cutting graphene sheets into narrow strips, (2) grow bilayer graphene (BLG) by Bernal stacking, and (3) form a heterojunction in graphene by introducing a material with a non-zero bandgap as a barrier.

##### Single Layer Graphene Field Effect Transistors

The lack of bandgap in the single layer graphene results in such transistors not being able to turn off. Therefore, they can only be used in the field of radiofrequency (RF) circuits. Wu et al. studied the formation of graphene FETs on SiC substrates. The cutoff frequency of the device with a gate length of 40 nm can be increased up to 350 GHz, which is much better than silicon transistors (40 GHz) under the same conditions [198].

##### Graphene Nanoribbons Field Effect Transistors

When the width of graphene nanoribbons is less than 10 nm, it exhibits semiconductor characteristics. The bandgap of GNRs is inversely proportional to the ribbon width. Studies have shown that, as the GNR width decreases, the on/off ratio of devices increases. Jiao et al. reported a plasma etching process to produce high quality, narrow GNRs with a bandgap of ~15 meV [199]. Wang et al. demonstrated sub-10 nm GNRs FETs with I_on_/I_off_ ratio up to 10^6^ and on-state current density as high as ~2000 μA/μm at room temperature [200]. 

Compared to large-area laminar graphene transistors, MOSFETs made from graphene nanoribbons have a significantly improved device current on/off ratio. If high-quality GNRs can be produced on a large scale in the future, such nanoribbon transistors will likely be applied to logic circuits in the future.

##### Bilayer Graphene Field Effect Transistors

If two graphene layers are asymmetrically stacked, so-called Bernal stacking, and a vertical electric field is applied to them, a bandgap can be generated in graphene. Zhou et al. determined the bandgap of graphene placed on SiC and the bandgap is 0.26 meV when there is only one graphene layer. As the number of layers increases, the bandgap will gradually decrease [201]. Liu et al. demonstrated a large number (>50) of dual-gated field-effect transistors with high on/off current ratios of 15 fabricated at random across the large-area bilayer CVD graphene film, which further confirms the quality of the synthesized graphene [202].

The BLG can be made to have a bandgap by the above method, but its value is small. These values so far are promising for the high mobility graphene application but is still insufficient for logic applications. The mobility of the carriers in the double-layered graphene structure is lower than that of the single-layer graphene without the bandgap.

##### Graphene Heterojunction Field Effect Transistors

In recent years, a heterojunction was formed on the surface and boundary of the graphene to form a heterojunction transistor by introducing a material with a non-zero bandgap as a barrier to generate a forbidden band. Graphene heterojunction transistors are generally classified into lateral heterojunctions and vertical heterojunctions. This method is a new research hotspot of graphene digital transistors. Moon et al. partially fluorinated the single-layer graphene to obtain a lateral heterojunction structure with a bandgap of about 2.93 eV and a current on/off ratio of 10^6^ [203]. 

Although it is still a great challenge to fabricate a dielectric material that is lattice-matched with graphene, the vertical heterojunction structure can construct a tunnel transistor and achieve a high current on/off ratio of >10^7^ [204]. In addition, graphene-based vertical heterostructures can be extended from FET electronics to optoelectronics, which is a promising direction.

In general, graphene field effect transistors (GFETs) still have a long way to go from practical applications. Although GFETs have broad application prospects, it also faces many challenges. In addition, other indicators for evaluating digital circuits, such as short channel effects, integration, and power consumption, should be considered.

#### 14.2.2. Graphene-Like Materials as Channel Materials

Transition-metal dichalcogenides (TMDCs), e.g., MoS_2_, WSe_2_, WS_2_, etc. have emerged as a new class of semiconductors that display distinctive properties at the monolayer thickness. They can be used in electronic devices such as transistors and in optical components as emitters and detectors. The band gaps of TMDC monolayers are in the visible range (between 400 nm and 700 nm). 

**Figure 32:**
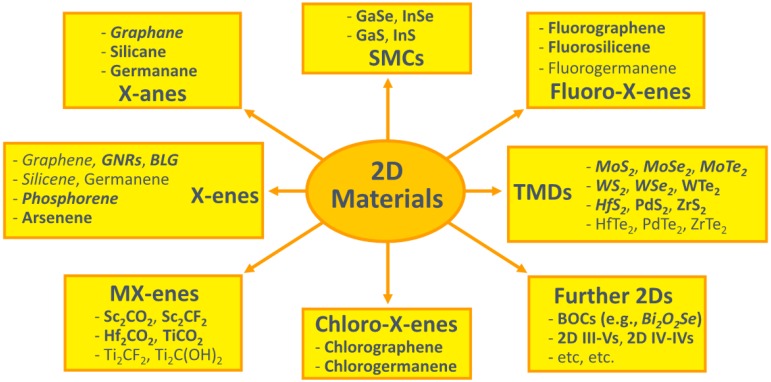
Drawing of different 2D materials [205].

Classes and representative examples of 2D materials together with representative materials for each group are shown in Figure 32 [205].

**Figure 33:**
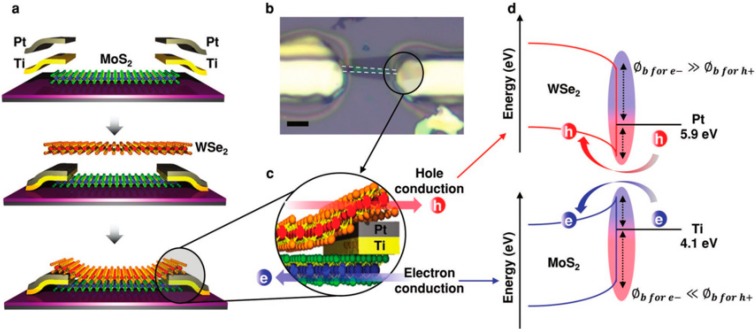
(**a**) Schematic of fabrication, a WSe_2_/MoS_2_ hetero-structure dual-channel FET. (**b**) Optical picture of a processed transistor. The dashed line shows the bottom MoS_2_ layer, (**c**) schematic of electron and hole transport in one channel of dual-channel FET, and (**d**) band diagram WSe_2_-Pt metal (top) and MoS_2_-Ti. The symbol ϕ_b_ in the picture stands for the barrier for hole and electrons [206].

A novel example for 2D transistors is shown in Figure 33. A dual-channel FET based on a vertically stacked hetero-structure of ultrathin n-type MoS_2_ and p-type WSe_2_ layers for the study of parallel carrier transport (electrons from MoS_2_ and holes from WSe_2_) have been demonstrated [206].

Atomic thin molybdenum disulfide (MoS_2_) is an ideal semiconductor material for field-effect transistors (FETs) with sub-10-nm channel lengths. The high effective mass and large bandgap of MoS_2_ minimize direct source-drain tunneling, while its atomic thin body maximizes the gate modulation efficiency in ultra-short-channel transistors. The sub-10 nm channel-length transistor was fabricated by directed self-assembly patterning of the mono-layer and tri-layer MoS_2_. This is done in a 7.5-nm half-pitch periodic chain of transistors where semiconducting (2H) MoS_2_ channel regions are connected to metallic-phase (1T) MoS_2_ and contact regions. The resulting 7.5-nm channel-length MoS_2_ FET has a low off current of 10 pA/µm, an on/off current ratio of >10^7^, and a subthreshold swing of 120 mV/dec.

**Figure 34:**
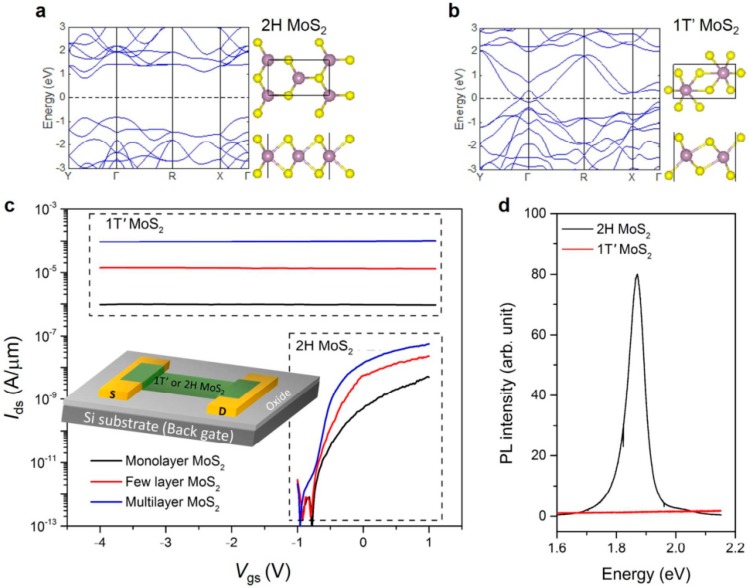
(**a**) and (**b**) Electronic band structures of 2H and 1T’ MoS_2_ and their atomic structures. The 2H band structure shows a bandgap of approximately 1.8 eV, while the conduction and valance bands of 1T’ MoS_2_ overlap. Therefore, 1T’ MoS_2_ has metallic gapless characteristics. (**c**) Transfer characteristics of three MoS_2_ FETs with different thicknesses of MoS_2_ before and after phase transition treatment. The intrinsic 2H MoS_2_ FETs show strong semiconducting behavior with large gate modulation, while the phase transition shows constant current with almost no gate modulation featuring, and (**d**) PL (photoluminescence)spectra of the monolayer 2H and 1T’ MoS_2_. The 2H phase shows a strong PL peak at 1.85 eV generated by its bandgap, while the PL of the 1T’ phase is completely quenched due to its gapless metallic characteristics [207].

To demonstrate and benchmark MoS_2_ transistors with channel lengths below 10 nm, two important challenges need to be overcome, which include a suitable lithography technology and a low-contact resistance for the S/D to ensure that the channel resistance will dominate the device behavior. To reduce the contact resistance, a junction between the metallic phase of MoS_2_ (1T) and its semiconducting phase (2H) has been used (see Figure 34) [207].

**Figure 35:**
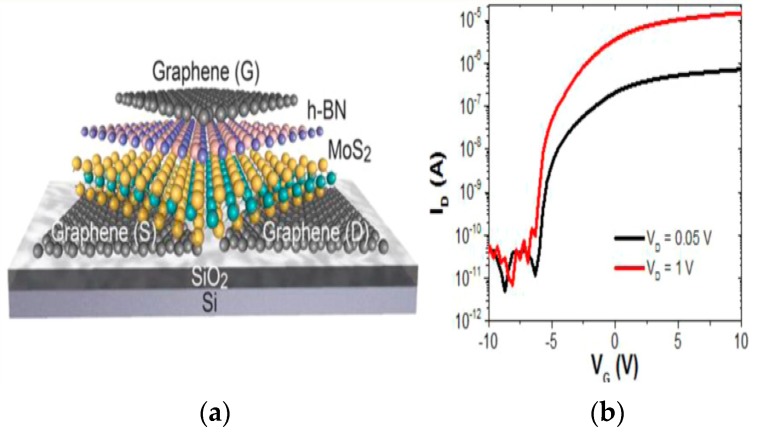
(**a**) Schematic of advanced 2D stacks and (**b**) characteristic curves of the transistor.

More complicated 2D material transistors can be realized by using heterogeneous stacks. For example, MoS_2_ is used as the active channel material and hexagonal-BN as the top-gate dielectric with graphene S/D (see Figure 35). This transistor exhibits n-type behavior with an ON/OFF current ratio of >10^6^, and an electron mobility of ∼33 cm^2^/Vs. The mobility does not degrade at high gate voltages, which presents an important advantage over conventional Si transistors where enhanced surface roughness scattering severely reduces carrier mobility values at high gate-fields [208].

(1) Tunnel Field Effect Transistors (TFET) 

The next generation of transistors in the future has to offer a sub-threshold swing of sub-60 mV/decade with a supply voltage < 0.6 V. In this field, different devices e.g., negative-capacitance FETs [209,210] and tunnel FETs (TFET) [211,212] are proposed. TFET is a gated p-i-n diode where the carriers are injected from the source to the channel region through the band-to-band tunneling (BTBT) mechanism [213]. Therefore, TFTs offer remarkably low I_OFF_ with a steep subthreshold slope. 

Since the sub-threshold swing decreases with the gate voltage bias, then the transistors have to be manufactured for a low voltage supply. In order to obtain high tunneling current and a steep slope, the transmission probability through the tunneling barrier has to be close to unity for a small variation of gate voltage. Therefore, the bandgap, the effective carrier mass, and the screening tunneling length have to be minimized for high barrier transparency. Finding appropriate materials for TFETs is an issue to be solved [214,215]. For example, Si-based TFETs have reported poor sub-threshold swing and low ON-current14 due to indirect band gap of 1.12 eV, which causes phonon-assisted tunneling (PAT) [216]. Recent studies suggest 2D materials have great properties for TFET channel material due to their planar structure and mechanical flexibility, outstanding electrostatic integrity, and small band gap with low effective mass. In this case, Graphene nanoribbon [217,218], transition metal di-chalcogenides (MoS_2_, WS_2_, MoSe_2_, WSe_2_, MoTe_2_, etc.) [219,220] Phosphorene [221,222], and group IV mono-chalcogenides (GeSe, GeS, SnSe, SnS) have been proposed for TFETs. Both hetero-bilayer [223,224] and hetero-junction transistors 25 are designed. TFT (field-effect transistors) technology is still not mature and needs more reliable fabrication techniques for mass production.

(2) New Devices in the Near Future

Intel has demonstrated a spintronic logic device, so-called magnetoelectric spin–orbit (MESO), which can be scaled down in energy per operation to a level of switching energy. This is 30 times below today’s CMOS transistors. MESO may operate at a voltage around 100 mV, which is 5 times below any advanced CMOS. The device functions by a ferroelectric/magnetoelectric switching and topological conversion of spin to charge. In addition, the non-volatility property offers remarkably low standby power, which is crucial for modern computing. MESO has the potential to be developed for multi-generational computing in the future [225].

## 15. Advanced Characterization for Ultra-Miniaturized CMOS

The continuous refinement of semiconductor manufacturing technologies urges the key sizes of devices to be down scaled while more challenges are created in testing methodologies. The expected measurements are not only focusing on critical dimension and thickness, but also on the 3D device structure on a nanometer scale. In research development in the future, it is necessary to measure the critical dimensions of the device structure, thickness of thin film, surface and interface properties, physical properties, and surface defects. It is expected that the test equipment should have a high-precision, high-speed, and is non-destructive, which can be used to monitor in-line. The application of advanced measurement strategies of integrated circuit critical dimension in ultra-miniaturized CMOS are presented below.

### 15.1. CD-SEM

Traditional SEM imaging is slow and cannot meet the needs of the semiconductor industry. High throughput SEM has a high detection speed and has been prepared and developed by processes below 10 nm. High throughput SEM technology is a general term for the integrated application of multiple technologies, including immersion rocker objectives lens, focus tracking technology, charge control technology, and high-speed image acquisition system. Their application makes large-area high-precision scanning imaging a reality, and, compared to imaging speed of the traditional SEM, is more than 300 times faster, where the low-voltage resolution is up to the nanometer level [226].

CD-SEM is an important test device in the front-end process of integrated circuits. CD-SEM is mainly used for in-line measurement of critical dimensions and performance monitoring of critical equipment during chip manufacturing. CD-SEM also plays an important role in optical proximity correction (OPC) model modification. Its main feature is the rapid and accurate automatic image recognition ability. In addition, CD-SEM can realize more test methods by constant optimization and improvement of the algorithm, such as Edge Roughness evaluation, Gap, wiggling, overlay, and center gravity. The advantages of CD-SEM makes the tool more useful in some form of continued applications. However, with the development of integrated circuits in the future, CD-SEM demands improving the resolution and assisting the modeling of OPC 2D graphics [227].

### 15.2. 3D AFM (Atomic Force Microscope)

3D AFM technology is based on a non-contact technique. In addition, this tool can be tilted at angles up to 38 degrees. AFM testing can be applied in the dimensional space, resolving sidewall and analyzing three-dimensional doping that most testing techniques cannot measure. 3D AFM has the nondestructive measurement of nano precision and accuracy, which can realize automatic analysis and cooperate with efficient production. 3D AFM technology can be used in In-line monitoring and engineering analysis. It overcomes the weakness of CD SEM in measuring CD at the bottom of the sidewall, and can test data more accurately than OCD (optical critical dimension), which saves the derivation work of the optical measurement [228].

### 15.3. 3D APT (Atom Probe Tomography)

The principle of elemental analysis by TEM/STEM is that an electron beam is required to act on the material and then elemental analysis is performed based on the information of the scattered electrons. At present, the elemental analysis results of TEM/STEM are basically two-dimensional. Due to the limitation of the resolution, for many elements with low atomic number, such as C, N, O, and Al, the resolution of TEM/STEM is low, and energy dispersive spectrometer (EDS), EELS elemental analysis cannot easily distinguish the elements [205]. The above reasons make TEM/STEM not provide the required information in some occasions. APT technology can overcome the problem to distinguish light elements, and directly obtain the element distribution in three-dimensional space in solid samples, which is a significant advantage compared to TEM/STEM technology. In most semiconductor devices, there are often multiple components, and their spatially ordered distribution constitutes nano-devices.

**Figure 36:**
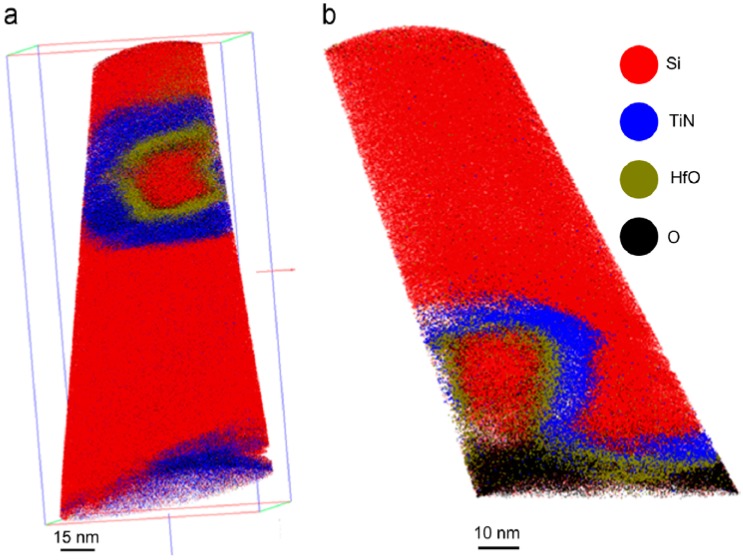
3D APT volume based on the standard reconstruction algorithm after density correction of GAA (**a**) and tri-gate (**b**) silicon nanowire transistor [229].

An element map with a high resolution can be obtained in space. Through the use of 3D Atom Probe Tomography (3D APT), the three-dimensional structure of the device can be characterized, and the parameters of the device as well as the size and spatial structure of each component can be understood. As shown in Figure 36, 3D APT volume is based on the standard reconstruction algorithm after density correction of GAA (a) and tri-gate (b) silicon nanowire transistor. An element map with a high resolution can be obtained in space. The interface of different materials is clearly visible, which can effectively help us understand the structural characteristics of these devices.

3D APT method reveals any non-uniform shape of the apex since the signals are distorted due to irregularity of nano-scaled shapes. In the future, we need to develop an advanced sample preparation technology that contributes for a better structure analysis but also better distinguish the corresponding positions of elements, which reduces the impact of material types. The interface states on resolution and avoiding defects leads to failure of the analysis results. APT also needs to combine more equipment to improve test results and analysis algorithms to restore the three-dimensional structure more realistically. This technology is still developing and improving in the semiconductor field [230,231].

### 15.4. Optical Critical Dimension

Optical critical dimension (OCD) measurement equipment is widely used in process development and process control with its fast, non-destructive, and non-contact testing methods. The principle is to obliquely illuminate the surface of the film with elliptically polarized light in a broadband band as well as to collect the reflection spectrum, and measure the thickness and width parameters of the three-dimensional structure by optical calculation. A spectral database is formed in the early stage and the collected measured spectra were fitted with the theoretical spectra to obtain the final results. The measurements could include the spectroscopic ellipsometry (SE), Mueller matrix (MM) SE, and normal incident polarized reflectometry, or multi-Angle multi-wavelength OCD [232,233].

Optical critical dimension measurement equipment is widely used in process development and process control with its fast, non-destructive, non-contact testing methods. The CD of the 3D device structure and the thickness of each film layer can be measured. However, OCD faces a new challenge as the size of the node becomes smaller. For example, the measurement is complicated for multi-film parameter measurements, 3D measurements, or to obtain a random structure parameter (roughness) in FinFET. In order to increase the sensitivity of the tool to different structural parameters such as CD or sidewall angle, extensive research has been conducted to use Mueller-matrix spectroscopic ellipsometry. In this field, using scatterometry as part of a hybrid metering scheme could help reduce parameter uncertainty [234,235,236].

### 15.5. Hybrid Metrology

The precision and high-efficiency test equipment has been remarkably improved for next-generations of transistor fabrication. In this field, hybrid metrology improves the metrology performance of complex device structures by combining different test methods. In this way, more functions can be performed and measurement errors are eliminated, which improves the accuracy of test results compared to a single test device.

For FinFET structures, in-line monitoring CD-SEM can measure CD well, but it is not sensitive enough to determine the fin’s height. Optical scattering can be used for FinFET structures, but relying on a large number of data for fitting, it greatly increases uncertainty in the measurement. 

The FinFET structure can be measured by the CD-SEM, AFM, or TEM. Then, the results are fed into an OCD tool to validate and to compare the measured data from different processes and to examine to which extent these techniques can detect subtle differences in the process [237]. It may be important to use a hybrid metering method to share information across technologies and to reduce uncertainty to an acceptable level. In the future, we may use atomized measurement equipment. The output can then be combined with in-plant metering such as OCD, AFM, and CDSEM to create a hybrid metering solution. Hybrid metrology and artificial intelligence can be integrated in the measurement and data analysis.

### 15.6. X-Ray Metrology Technologies 

Film metrology is an important issue besides CDs. There are three challenges in the metrology arena: compositional, dopant, and strain. 

When the technology node moves toward 5 nm, the traditional metrology techniques maybe touch their limits [45]. FinFET and GAA structures create a need for 3D metrology where X-ray plays a significant role by using the following technologies- XRF (X-ray fluorescence), XPS (X-ray photoelectron spectroscopy), XRR (x-ray reflectivity), XRDI (X-ray diffraction image), XRD (X-Ray Diffraction), HRXRD (high resolution X-Ray Diffraction), LEXES (low energy electron induced X-ray emission spectrometry), CD-SAXS, and GISAXS.

XRF is a photometric technique, which is used to look at surface contamination. The XRF/XPS combo tool is applied to determine the composition and chemistry on the surface. XRR handles thin films and XRDI is used to detect wafer level defects such as slip.

HRXRD, or XRD, is a technique to characterize composition, thickness, dopant concentration and strain in devices [238,239,240,241,242,243]. HRXRD is used to qualify the repeating patterns of FinFETs [244,245,246,247]. The LEXES is involved for dopants in materials.

HRXRD is emphasized as an important tool to characterize 10-nm node and beyond. It has been demonstrated that in-line HRXRD can monitor the pre-fin and post-fin etching processes in FinFET [248]. IMEC demonstrated an in-line HRXRD set-up for analyzing composition and strain for nano-scale level devices. They have successfully studied the composition and strain state of etched and selectively grown Ge/SiGe fins as well as multilayer fin width down to 16 nm. The RSM (reciprocal space mapping) of (113) reflection acquired from fins, which provides information about the lattice parameters in two directions, and calculate fin pitch, according to the spacing between first order grating rods in the figure [247]. It is important to mention that HRXRD is also used to characterize III-V fins in sub-10 nm as well.

**Figure 37:**
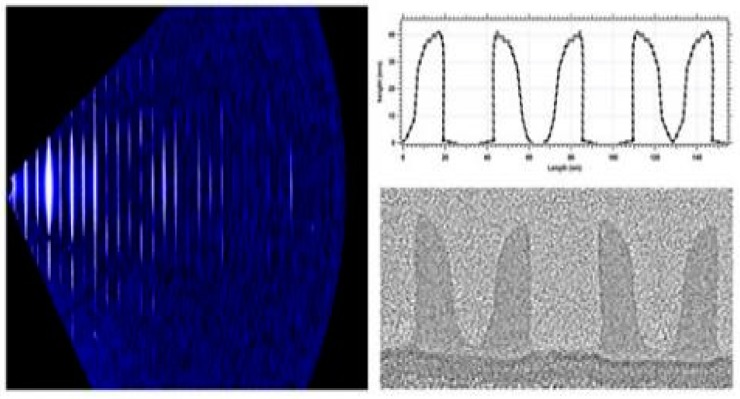
RSM and fits to the scattering profile of samples [250].

Beyond metrology for epitaxy application, the semiconductor industry is also exploring new metrology techniques for future requirement to characterize a three-dimensional structure, where the critical dimensions are less than 10 nm. CD small-angle X-ray scattering (CD-SAXS) is non-destructive and a promising technique to characterize the nano-devices [249,250,251]. This technique collects a series of scattering signals by a small rotation angle of samples. The signals contain both in-plane and out-of-plane information. This technique is used to reconstruct the 3D reciprocal-space for measuring line profiles of short grating, as shown in Figure 37. CD-SAXS provides real potential solutions for replacing the traditional techniques like CD-SEM and OCD and it is applicable to any type of material-crystalline, polycrystalline, and resists. 

Intel and the National Institute of Standards and Technology (NIST) have demonstrated CD-SAXS measurements from patterned 12 nm lines device with 0.5 nm spacing [250]. Using CD-SAXS, an accuracy down to 0.1 nm has been reported. CD-SAXS can be used for 7 nm or 5 nm structures for 3D memory, advanced EPI (epitaxy), and FinFETs with no calibration.

**Figure 38:**
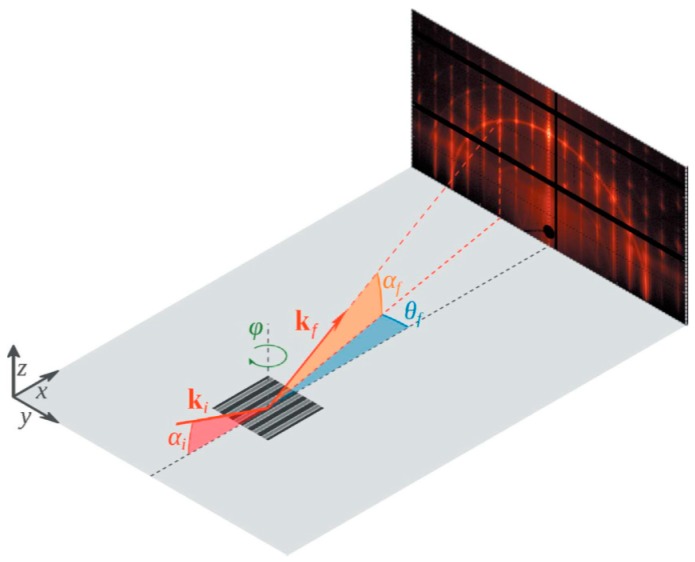
Geometry of GISAXS experiments [252].

Another strategy for measuring critical dimensions is using Grazing-incidence small-angle X-ray scattering (GISAXS) [252]. The measurement geometry of GISAXS is shown in Figure 38 GISAXS can measure small targets, which are sensitive to the grating line profile. The technique shows the ability to extract structural parameters of the gratings depending on scanning the photon energy and the scattering intensity.

### 15.7. Artificial Intelligence in Metrology

Artificial Intelligence in complex software programs is also involved in metrology recently. Machine learning is an emerging technique, which will not replace metrology tools, but it could help solve the most difficult metrology challenges at 10 nm node technology and beyond. The companies—Nova launched its Artificial Intelligent software NOVAFit™, using machine learning as a complementary method to predict the fin CD values from in-line measurements [253]. By using such a technique, the electrical resistance in interconnects using data from both OCD and electrical tests could be predicted. The new software program improves metrology capabilities and accelerates time to analyze complex 3D devices with a high aspect ratio.

## 16. Conclusions

This article has presented the principle of miniaturization of MOSFET and a survey of technology roadmap for CMOS. An overview of transistor processing with a focus on the approach to the end has been provided.

The discussions initiated from state-of-art lithography of nano-scale patterns using the extreme ultraviolet (EUV) lithography and 193 nm immersion with multi-patterning. Even though EUV simplifies the patterning process for the 7 node, EUV still has issues with resists and mask infrastructure as well as power source, which have to be solved before high-volume manufacturing.

Furthermore, the challenges of FinFET processing and their relations to electrical characteristics were discussed.

In FinFETs, high current should be transported in fins. Therefore, longer fins are required. However, there is a difficulty for integration of poly-gate, spacer, and the replacement metal gate. For example, to etch the poly-gate with a high aspect ratio, it suffers from charging and micro-loading of etching, which results in variable gate length. It is concluded that an optimum wet and dry etch at low temperature and low etch rate is needed to avoid gate length variation and Si loss in fins.

SiGe has used as stressor material for source/drain regions starting from a 90 nm technology node. In order to have uniform SiGe epitaxy, a uniform chip layout is needed to minimize the pattern dependency of the growth. 

In approach to the end of the technology roadmap, FinFETs become lateral or vertical nanowire transistors (LGAA or VGAA, respectively). In VGAA, the nanowire contains SiGe/Si or SiGe/Ge stack where Si, Ge, or SiGe can act as the channel of the transistor. Si or SiGe material could be selectively etched by using TMAH solution mixed by ACT^®^ SG-201.

In the nanowire transistors, traditional ion implantation cannot be used and new doping strategies e.g., monolayer and plasma doping are required to dope the nanowires.

After planar transistors were changed to 3D FinFETs in 22-nm node, HfO_2_ has been accepted as high-*k* material due to its high dielectric constant and a relatively large bandgap. However, the integration of HfO_2_ in nano transistors has a problem of the thermal instability of HfO_2_/Si interface. The SiO_x_ interlayer between HfO_2_ and Si substrate can improve the interfacial imperfection. The thickness of this SiO_x_ interlayer and the high-*k* dielectric have been continuously decreased in each new technology node. Si polycrystalline gate-material was also abandoned and metal-gates like TiAlN and TiN were introduced in a gate-last approach to prevent crystallization of the high-*k* material during the thermal treatments. 

In integrated circuits, ALD W with α-phase is mainly applied as electrode filling. One way to grow tungsten films with α-phase on SiO_2_ is to use WF_6_ as a precursor and H using hot-wire (HW) assisted atomic layer deposition (HWALD).

Cu is a classical interconnect material microelectronic circuit. However, as the CD narrows, filling the BEOL trench-over-via structure by Cu becomes more challenging. Introducing Co via the prefill concept makes it possible for void-free and bottom-up fill of metal in advanced interconnects. Co is expected to have a better EM performance compared to Cu due to its higher melting point.

Reliability test over of a processed wafer is very important for IC manufacturers and this becomes more critical when miniaturization of CMOS occurs. The checking points are novel materials, novel process, and novel integration where the reliability characterization provides information about the physical mechanism of degradation.

The random telegraph noise is also an issue that becomes more important for nano-scaled transistors and could act as an indicator for transistor performance. As an example, the self-heating in transistors could be a source for the signal noise. 

In the future, when the Beyond Moore era is reached, it is believed new material, e.g., III-V and 2D crystals will be insightful. The main problem is integration of these materials with high quality on Si. So far, III-V devices, e.g., InGaAs-OI FinFET, InGaAs FET, III-V Tunnel FETs (TFETs), and hybrid InGaAs/SiGe CMOS on a silicon substrate by using advanced template-assisted selective epitaxy technology. 

Among 2D material Graphene, transition-metal dichalcogenides (TMDCs), e.g., MoS_2_, WSe_2_, and WS_2_ have a strong position for devices. Since the 2D material have a lack of bandgap, then three methods are proposed. The methods include narrow strips, bilayers, and heterojunction in order to create a bandgap. 

Many promising devices from 2D materials have been manufactured. As an example, a dual-channel FET based on a vertically stacked hetero-structure of ultrathin n-type MoS_2_ and p-type WSe_2_ layers for the study of parallel carrier transport (electrons from MoS_2_ and holes fromWSe_2_) have been demonstrated.

More complicated 2D material transistors can be realized by using heterogeneous stacks. For example, MoS_2_ is used as the active channel material and hexagonal-BN as the top-gate dielectric with graphene S/D. This type of transistors exhibit n-type behavior with an ON/OFF current ratio of >10^6^, and an electron mobility of ∼33 cm^2^/Vs. The mobility does not degrade at high gate voltages, which presents an important advantage over conventional Si transistors where enhanced surface roughness scattering severely reduces carrier mobility values at high gate-fields.

In the research development in future production, it is necessary to measure the critical dimensions of the device structure, thickness of thin film, surface and interface properties, physical properties, and surface defects. It is expected that the test equipment should have a high-precision, high-speed, and be non-destructive, which can be used to monitor in-line. The famous tools are CD SEM, OCD, and 3D AFM, which can be used for the three-dimensional structure of the device. Recently, a new technique 3D APT, which provides an element map with a high resolution has been developed. In this way, the three-dimensional structure of the device can be characterized.

It is also required to use a combination of these techniques, which could provide information about more complex device structures.

A series of x-ray techniques, e.g., XRF, XPS, XRR, XRDI, XRD, HRXRD, LEXES, CD-SAXS, and GISAXS are also used for in-line measurements. These techniques provide information about crystalline materials for composition, thickness, dopant concentration, and strain in devices.

Recently, Artificial Intelligent in a new software form is also involved in metrology, which can help solve the most difficult metrology challenges at 10-nm node technology and beyond. The new software improves metrology capabilities and accelerates time to solution in complex 3D and High Aspect Ratio devices.

## Figures and Tables

**Table 1:** Material selection of high-*k* dielectric and metal gate from 45-nm to 5-nm nodes.

Technology Nodes	Film Thickness (nm)			
	Thermal Oxide	High-*k*	TiAl(N)	TiN
45 nm	~1.2	~1.5	~2	~2.1
32 nm	~1.2	~1.1	~1.7	~2
22 nm	~1.1	~1.0	~1.2	~1.4
14 nm	~0.6	~1.2	~1.2	~1.4
5 nm	~0.5	~1.0	~1.0	~1.2

**Table 2:** The effective work function of different metals grown by ALD for NMOSFET.

Metal	Dep. Method	Effective Work Function	Ref.
TaC_y_	PEALD	4.77–4.54 eV	[84]
TaCN	PEALD	4.37 eV	[85]
TiC	PEALD	5.24 eV–4.45 eV	[86]
WC_0.4_	PEALD	4.2+/-0.1 eV	[87]
ErC_2_	ALD	3.9 eV	[88]
TiAlC	thermal ALD	4.79–4.49	[90]
TiAlC	thermal ALD	4.46–4.24	[91]
TaAlC	thermal ALD	4.74–4.49	[92]
TaAlC	thermal ALD	4.65–4.26	[93]

**Table 3:** Summary of all MOL Metallization Options [158].

No.	Process Name	Material		Normalized Total Resistance
		S/D Contact Level	MOL LI Level	
1	By Scaling	Ti/TiN W	Ti/TiN W	1
2	Process A	Ti/TiN W	Co	0.85
3	Process B	Ti/TiN W	Liner free W	0.7
4	Process C	Liner free W	Co	0.55
5	Process D	Liner free W	Ru	0.55
